# Risk and protective factors for coronavirus disease 2019 (COVID-19) in allergic rhinitis patients: a national survey in China

**DOI:** 10.3389/falgy.2024.1479493

**Published:** 2024-12-10

**Authors:** Xiaozhe Yang, Yutong Sima, Jinming Zhao, Jing Zhang, Xiangdong Wang, Luo Zhang

**Affiliations:** ^1^ Department of Otolaryngology Head and Neck Surgery, Beijing Tongren Hospital, Capital Medical University, Beijing, China; ^2^ Department of Allergy, Beijing Tongren Hospital, Capital Medical University, Beijing, China; ^3^ Beijing Laboratory of Allergic Diseases, Beijing Municipal Education Commission and Beijing Key Laboratory of Nasal Diseases, Beijing Institute of Otolaryngology, Beijing, China; ^4^ Research Unit of Diagnosis and Treatment of Chronic Nasal Diseases, Chinese Academy of Medical Sciences, Beijing, China

**Keywords:** allergic rhinitis, COVID-19, risk factor, protective factor, infection

## Abstract

**Background:**

Several epidemiological studies have shown that allergic rhinitis (AR) patients are more susceptible to coronavirus disease 2019 (COVID-19).

**Objective:**

We aim to investigate the risk factors for COVID-19 in AR patients.

**Methods:**

A retrospective nationwide cohort study was conducted based on a questionnaire survey in China. The baseline characteristics, region of residence, smoking and drinking status, comorbidities, vaccination status and previous infection information were obtained. Allergen test results, the SARS-CoV-2 nucleic acid test, and antigen detection results were collected. Information on AR and comorbid medication use pre-SARS-CoV-2 infection was also collected. Binary logistic regression and analysis of covariance (different adjusted models) were conducted.

**Results:**

In all, 830 AR patients were included; 627 patients (75.54%) were infected with SARS-CoV-2. AR comorbid with allergic conjunctivitis (AC) was a protective factor [OR: 0.525 (95% CI = 0.296–0.929), *P* = 0.027] against SARS-CoV-2 infection, while AR comorbid with food allergy was a risk factor [OR: 6.404 (95% CI = 1.349–30.402), *P* = 0.0195]. Although fewer patients received four doses of the vaccine, the results showed a significant protective effect against SARS-CoV-2 infection in AR patients [OR: 0.093 (95% CI = 0.025–0.348), *P* = 0.0004]. Underweight was a protective factor against COVID-19 [OR: 0.287 (95% CI = 0.147–0.562), *P* = 0.0003] after full multivariable adjustment. Overweight was associated with a 2.071-fold higher risk for COVID-19 compared with normal weight [(95% CI = 1.045–4.105), *P* = 0.0370]. Additionally, house dust mite (HDM)-specific allergies were also protective against COVID-19 [OR: 0.537 (95% CI = 0.290–0.996), *P* = 0.0484].

**Conclusions:**

This study revealed underlying protective and risk factors, which might be used to improve the management of AR and COVID-19.

## Introduction

Severe acute respiratory syndrome coronavirus-2 (SARS-CoV-2), which causes coronavirus disease 2019 (COVID-19), has had a huge impact on global population health. According to the Johns Hopkins Coronavirus Resource Centerboard (https://coronavirus.jhu.edu/map.html), there have been more than 600,000,000 COVID-19 cases and 687,000 related deaths globally as of March 1, 2023. More COVID-19 cases are probably not diagnosed due to limited testing availability (1). With the rapid increase in the number of patients with COVID-19, clinical progress and prognosis have received more attention. Allergic disease is associated with complex immune responses (2), and persistent inflammation may affect the structure and abnormalities in their function (3). Allergic rhinitis (AR) is the most common nasal disorder, which contributes to the aggravation of respiratory virus-related disease (4) and affects 10%–40% of the population (5, 6). Interestingly, there was no statistically significant difference between the incidence of AR diagnosed during the pandemic compared with the period before the pandemic (7). The incidence of AR comorbid with COVID-19 was 1.8% in China (8). Whether AR is a risk factor for COVID-19 is controversial; nevertheless, accumulating evidence suggests that AR is a risk factor for COVID-19. In a nationwide Korean cohort study, AR conferred a greater risk of sensitivity to SARS-CoV-2 infection and severe clinical outcomes (9). In a Chinese cohort study, AR was also an independent risk factor [OR: 1.324 (95% CI: 1.049–1.671), *P* = 0.0183] for susceptibility to COVID-19 based on a propensity-score-matched nationwide cohort (10). However, a separate study in China showed that AR may not have a significant modifying effect on the development of COVID-19 (11). Moreover, AR patients had lower positive SARS-CoV-2 test rates in a cohort study in England (12). Even in a study in Iranian patients, AR disease status was inversely associated with the severity of COVID-19 (13). Based on clinical observations, AR patients have shown diverse clinical manifestations during the SARS-CoV-2 pandemic. Considering the current extent of different effects of AR disease status on COVID-19 and the complex pathological mechanisms of AR, we further investigated the risk factors for COVID-19 in AR patients.

China adjusted the national prevention and control strategy of 2019-nCoV infection and emerge an epidemic peak from early Dec.2022. We immediately and efficiently performed a nationwide survey with large sample size and short collection time (Dec. 28, 2022-Jan. 13, 2023), and the survey time coincided with this epidemic peak. To our knowledge, this is the first nationwide cohort study exploring the risk factors for COVID-19 in AR patients, with the goal of improving the management of AR and COVID-19.

## Methods

### Study population

**Figure 1:**
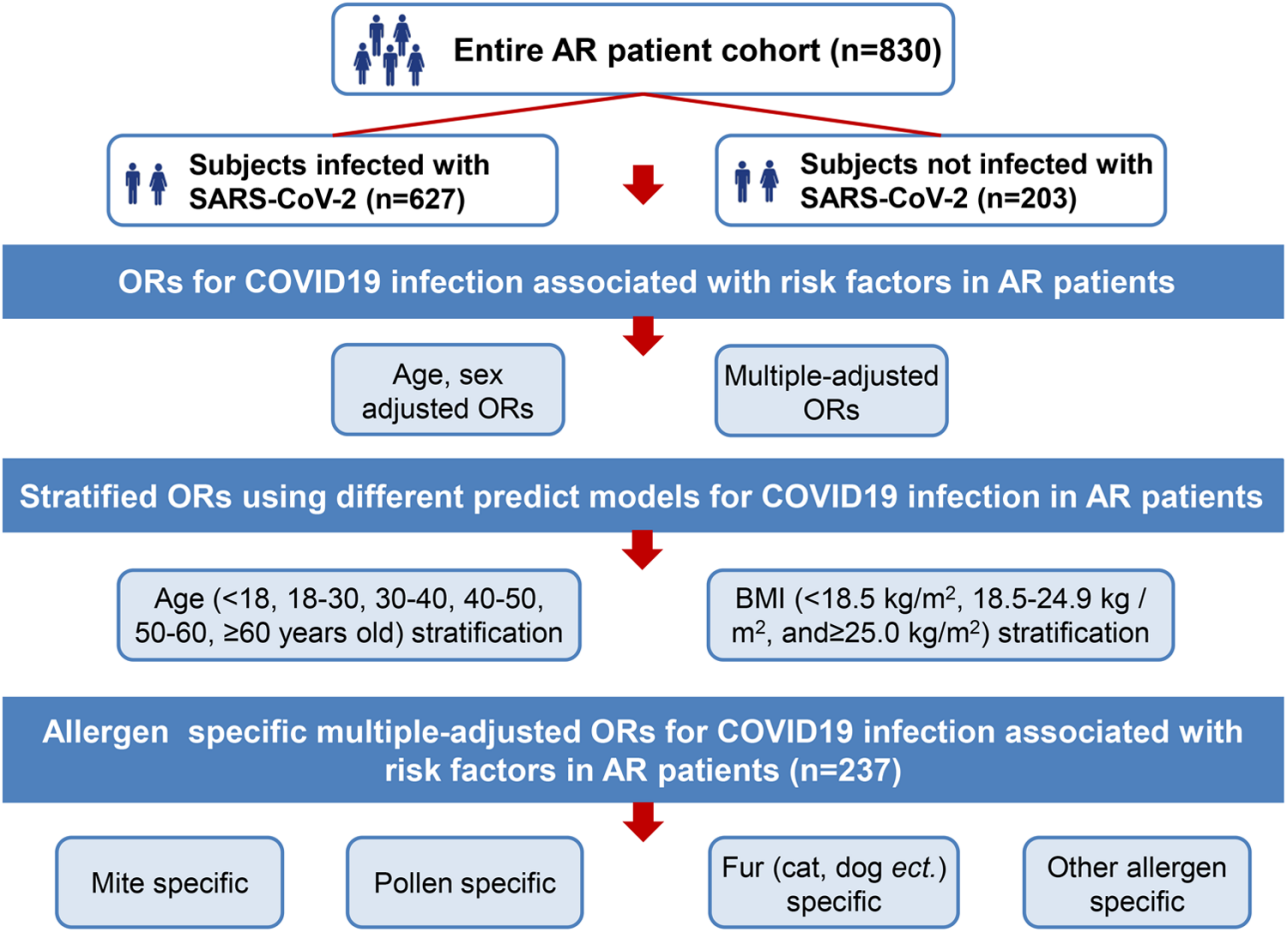
Study enrollment and data analysis.

This was a retrospective cohort study. A structured questionnaire encompassing various aspects of AR and COVID-19 results was designed. We conducted a structured questionnaire survey using convenience sampling. An online informed consent form was provided to participants. The questionnaire was self-administered and was required to be completed online (mini app in WeChat on mobile phone) by the participating volunteers. At the start of the survey, all participants were told about the study goals. Since it was an anonymous questionnaire, personal information was not recorded. This study was conducted following the Declaration of Helsinki (1964) guidelines and its later amendments or comparable ethical standards. Participation in the survey was voluntary. A total of 830 participants were involved the questionnaire survey during the period (Dec. 28, 2022∼Jan. 13, 2023) (Figure 1). The online questionnaire was set so that each IP address could only answer once to avoid duplicate answers and questionnaires with missing data cannot be submitted. Our study followed the STROBE guideline, STROBE checklist was provided as Supplementary Material.

### Measurement

The characteristics of age, sex, weight, height, BMI, region of residence, smoking and drinking habits, comorbidity, vaccination status, previous infection with SARS-CoV-2 or not, and so on were collected. Information on allergen test results [mite, pollen, fur allergy (cat, dog, *etc.*) and other allergens] was collected. The questionnaire also included items related to the SARS-CoV-2 nucleic acid test and antigen detection results. Information on AR and comorbidity medication use pre-SARS-CoV-2 infection was also collected (Figure 1).

### Statistical analysis

The sample size of 609 subjects was calculated based on the estimated prevalence of COVID-19 infection prevalence of 72.49%, to attain a significant level (alpha) of 0.05 and error tolerance 0.10 *p* (or 10%) using PASS15 software (NCSS, USA).

Baseline characteristics are presented as the mean ± (SD)/median (min, max) and the number (%) for continuous and categorical variables, respectively. The *t* test/Satterthwaite's test (equal variances not assumed)/paired *t*-test, chi-square (*χ*^2^) test/Fisher's exact test, or Wilcoxon rank-sum test was conducted to compare participant characteristics between two groups.

We performed multivariable logistic regression analyses to investigate risk factors for COVID-19 in AR patients. Five logistic regression models (OR^1–5^) including different confounding factors were involved in our analysis. We also calculated BMI stratification (underweight, normal weight, and overweight) and age stratification and allergen specific ORs for COVID-19 in AR patients as sensitive analysis.

All statistical analyses were performed with SAS (version 9.4). A *p* value < 0.05 (two-sided) was set as statistically significant.

### Analysis overview

The following analyses were performed:

(1) Demographic and clinical characteristics and SARS-CoV-2 infection results of all study subjects (*n* = 830) were compared (infected with SARS-CoV-2 *v.s.* not infected with SARS-CoV-2).
(2) We conducted a primary analysis using binary logistic regression and analysis of covariance (model adjusted for age and sex; fully adjusted for all covariance) to control potential confounding factors and calculate the odds ratio (OR) and 95% confidence interval (95% CI) of outcomes.
(3) Age stratification (younger than 18 years old, 18–30, 30–40, 40–50, 50–60, and older than 60 years old) and BMI stratification (<18.5 kg/m^2^, 18.5–24.9 kg/m^2^, and ≥25.0 kg/m^2^) ORs for COVID-19 in AR patients (*n* = 830) were calculated based on different adjusted models (model 1, adjusted for age, sex; model 2, adjusted for age, sex, weight, height; model 3, adjusted for age, sex, weight, height, BMI, region of residence, education, occupation, smoking and drinking; model 4, adjusted for age, sex, weight, height, BMI, region of residence, education, occupation, smoking and drinking, comorbidity, treatment; model 5 (fully adjusted), adjusted for age, sex, weight, height, BMI, region of residence, education, occupation, smoking and drinking, comorbidity, treatment, vaccination and previous infection).
(4) Allergen [mite, pollen, fur allergy (cat, dog *etc.*), and other allergen]-specific multiple-adjusted ORs for COVID-19 (*n* = 237) were calculated in AR patients (patients who had allergen testing results).
(5) We redefined the cohort by using a stricter definition of COVID-19 status as self-reported COVID-19 and PCR-test positivity or antigen detection positivity or both (Supplementary Materials). Then, we performed the primary analysis again to calculate the ORs for COVID-19.

## Results

### Demographics and clinical characteristics

A total of 830 patients with AR and available data were included in this study, and the demographic and clinical characteristics are summarized in Table 1. In this cohort, 627 patients (75.54%) had AR comorbid with SARS-CoV-2 infection, and 203 patients (24.46%) did not. In these two groups, patients' age, sex, BMI, region of residence, education attainment, occupation, and smoking or drinking habits were not significantly different. Considering allergic disease comorbidities, the number of patients with AR comorbid with allergic conjunctivitis (AC) and AR comorbid with food allergy was relatively lower than the number of AR patients without these comorbidities (*P* = 0.0300 and *P* = 0.0048, respectively). Other comorbidities, including chronic rhinosinusitis (CRS), asthma, bronchitis, and skin allergy, did not show variation. Patients received vaccines at different frequencies, with the majority of patients receiving 3 doses of vaccine (*P* < 0.0001). Most patients had no history of previous SARS-CoV-2 infection. Patients who received medication for AR or other comorbidities also did not show differences.

**Table 1:** Demographic and clinical characteristics and SARS-CoV-2 infection results of all study subjects.

Characteristic	Entire cohort (*n* = 830)	SARS-CoV-2 (*n* = 627)	Non-SARS-CoV-2 (*n* = 203)	*t* or *χ*^2^/*P* value
Age (y), mean ± SD	35.03 ± 11.40	34.79 ± 11.21	35.78 ± 11.95	-1.07/0.2838
Median (min, max)	34 (5, 88)	34 (5, 87)	35 (6, 88)	
Sex, *n* (%)				
Male	355 (42.77)	262 (31.57)	93 (11.20)	1.0157/0.3135
Female	475 (57.23)	365 (43.98)	110 (13.25)	
Height, mean ± SD	166.5 ± 9.62	64.05 ± 12.86	63.10 ± 13.19	0.91/0.3649
Weight, mean ± SD	63.82 ± 12.94	166.3 ± 9.65	166.9 ± 9.54	−0.84/0.4033
BMI, mean ± SD	22.92 ± 3.81	23.06 ± 3.88	22.4788 ± 3.55	1.90/0.0574
Region of residence, *n* (%)				
Urban	717 (86.39)	539 (64.94)	178 (21.45)	0.3857/0.5346
Rural	113 (13.61)	88 (10.60)	25 (3.01)	
Education attainment, *n* (%)				
Primary school and lower	23 (2.77)	15 (1.81)	8 (0.96)	3.3241/0.1897 for trend
Middle and high school	135 (16.27)	96 (11.57)	39 (4.70)	
College and higher	672 (80.96)	516 (62.17)	156 (18.80)	
Occupation, *n* (%)				
Healthcare	36 (4.34)	29 (3.49)	7 (0.84)	1.4225/0.4910 for trend
Teaching/student	144 (17.35)	104 (12.53)	40 (4.82)	
Other	650 (78.31)	494 (59.52)	156 (18.80)	
Smoking status, *n* (%)				
Never	652 (78.55)	494 (59.52)	158 (19.04)	0.4346/0.8047 for trend
Previous	38 (4.58)	27 (3.25)	11 (1.33)	
Current	140 (16.87)	106 (12.77)	34 (4.10)	
Drinking status, *n* (%)				
Never	495 (59.64)	367 (44.22)	128 (15.42)	1.3097/0.5195 for trend
Previous	14 (1.69)	11 (1.33)	3 (0.36)	
Current	321 (38.67)	249 (30.00)	72 (8.67)	
Comorbidity				
CRS, *n* (%)				
Yes	110 (13.25)	80 (9.64)	30 (3.61)	0.5438/0.4609
No	720 (86.75)	547 (65.90)	173 (20.84)	
Asthma, *n* (%)				
Yes	28 (3.37)	20 (2.41)	8 (0.96)	0.2654/0.6064
No	802 (96.63)	607 (73.13)	195 (23.49)	
Bronchitis, *n* (%)				
Yes	45 (5.42)	34 (4.10)	11 (1.33)	0.0000/0.9983
No	785 (94.58)	593 (71.45)	192 (23.13)	
Allergic conjunctivitis, *n* (%)				
Yes	68 (8.19)	44 (5.30)	24 (2.89)	4.7074/0.0300*
No	762 (91.81)	583 (70.24)	179 (21.57)	
Skin allergy, *n* (%)				
Yes	181 (21.81)	143 (17.23)	38 (4.58)	1.5028/0.2202
No	649 (78.19)	484 (58.31)	165 (19.88)	
Food allergy, *n* (%)				
Yes	38 (4.58)	36 (4.34)	2 (0.24)	7.9413/0.0048**
No	792 (95.42)	591 (71.20)	201 (24.22)	
Vaccination frequency, *n* (%)				
0	36 (4.34)	27 (3.25)	9 (1.08)	34.6048/<.0001* for trend
1	28 (3.37)	16 (1.93)	12 (1.45)	
2	149 (17.95)	117 (14.10)	32 (3.86)	
3	597 (71.93)	462 (55.66)	135 (16.27)	
4	20 (2.41)	5 (0.60)	15 (1.81)	
Previous SARS-CoV-2 infection, *n* (%)				
0	784 (94.46)	596 (71.81)	188 (22.65)	4.2725/0.2335 for trend
1	42 (5.06)	27 (3.25)	15 (1.81)	
2	3 (0.36)	3 (0.36)	0 (0.00)	
≥3	1 (0.12)	1 (0.12)	0 (0.00)	
Medication for AR or comorbidities				
Yes	386 (46.51)	337 (40.60)	107 (12.89)	0.0665/0.7965
No	444 (53.49)	290 (34.94)	96 (11.57)	

Numbers in bold face indicate significant differences (*P* < 0.05). *P < 0.05, **P < 0.01, ***P < 0.001.

We also analyzed AR patients under a stricter definition of COVID-19, where in these patients had PCR test positivity, antigen detection positivity, or both of them. Under this definition, there were 460 (55.42%) patients with AR comorbid with COVID-19 and 370 (44.58%) patients with AR without COVID-19 comorbidity (More details are given in Supplementary Table S1).

### Effect of demographics and clinical characteristics on COVID-19

**Figure 2:**
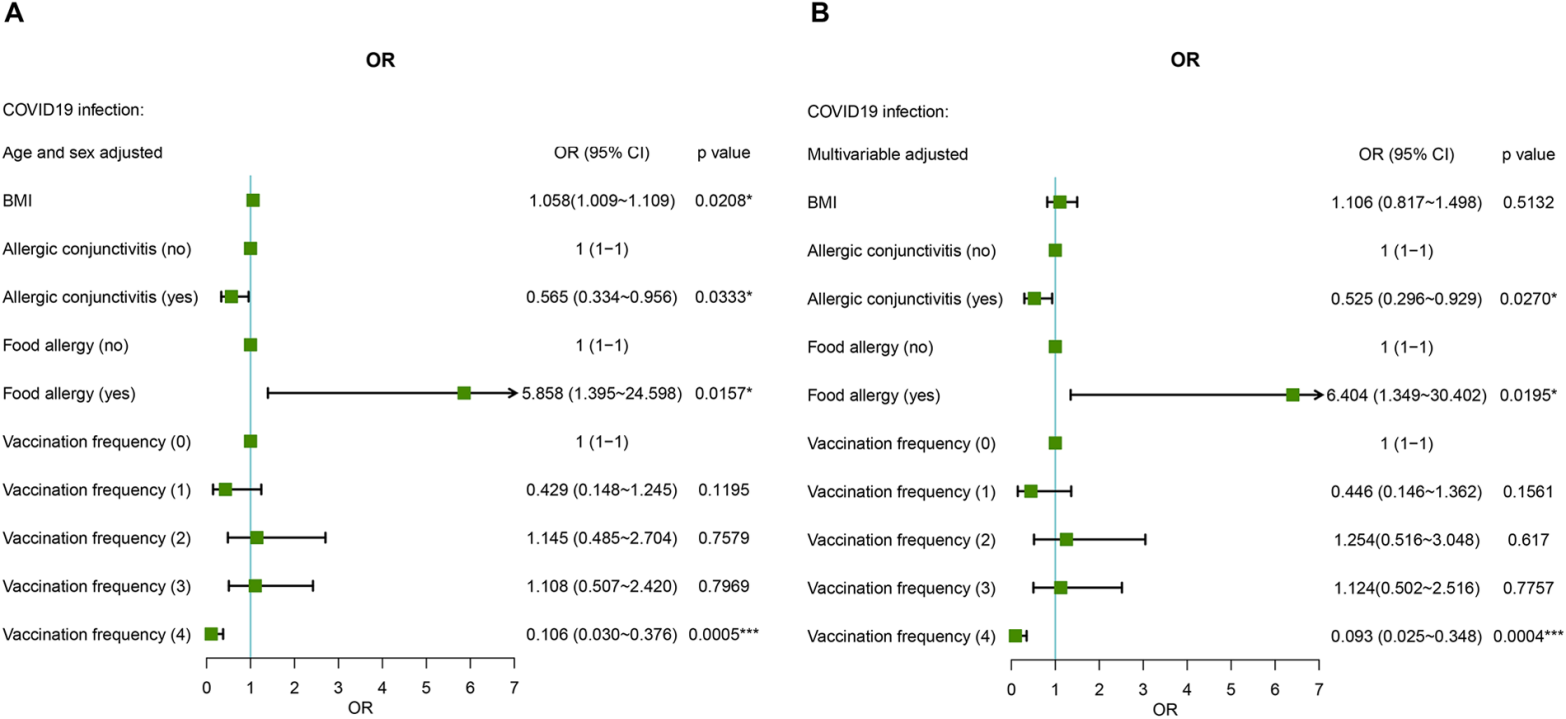
Age-, sex- **(A)** and multiple-adjusted **(B)** ORs for COVID-19 associated with risk factors in AR patients.

BMI was a risk factor for COVID-19 (OR: 1.058 (95% CI = 1.009–1.109, *P* = 0.0208) after adjusting for age and sex in the AR cohort. After full multivariable adjustment, BMI was not significantly associated with COVID-19 (*P* = 0.4275). The region of residence, educational attainment, occupation, smoking habits, and drinking habits of AR patients were not related to COVID-19 after age and sex adjustment or full multivariable adjustment (all *P* values > 0.05) (Figure 2 and Table 2).

**Table 2:** Multiple-adjusted ORs for COVID-19 infection associated with risk factors in AR patients.

Characteristic	Age, sex adjusted		Multivariable adjusted^a^	
	OR [95% CI]	Wald χ^2^/*P* value	OR [95% CI]	Wald χ^2^/*P* value
Age	0.992 [0.979–1.006]	1.2358/0.2663	0.986 [0.971–1.002]	2.8634/0.0906
Sex				
Male	0.843 [0.613–1.160]	1.1006/0.2941	0.917 [0.548–1.533]	0.1101/0.7401
Female (ref 1.0)				
Height	0.998 [0.977–1.019]	0.0509/0.8215	0.998 [0.921–1.081]	0.0030/0.9561
Weight	1.014 [0.999–1.028]	3.2863/0.0699	0.980 [0.877–1.094]	0.1345/0.7138
BMI	1.058 [1.009–1.109]	5.3441/0.0208*	1.106 [0.817–1.498]	0.4275/0.5132
Region of residence				
Urban	0.910 [0.560–1.478]	0.1461/0.7022	0.927 [0.550–1.564]	0.0801/0.7771
Rural (ref 1.0)				
Education attainment		3.5352/0.1707 for trend		2.8458/0.2410 for trend
Primary school and lower (ref 1.00)				
Middle and high school	1.441 [0.548–3.790]	0.5491/0.4587	1.671 [0.487–5.737]	0.6656/0.4146
College and higher	1.913 [0.777–4.711]	1.9888/0.1585	2.207 [0.670–7.277]	1.6923/0.1933
Occupation		2.4471/0.2942 for trend		2.9514/0.2286 for trend
Teaching/student (ref 1.0)				
Healthcare	1.742 [0.700–4.332]	0.8641/0.3526	2.171 [0.780–6.044]	1.5825/0.2084
Other	1.350 [0.884–2.061]	0.0080/0.9285	1.372 [0.870–2.163]	0.0666/0.7964
Smoking status		0.2870/0.8663 for trend		0.8367/0.6581 for trend
Never (ref 1.0)				
Previous	0.887 [0.421–1.870]	0.0986/0.7535	0.698 [0.315–1.547]	0.7840/0.3759
Current	1.091 [0.692–1.722]	0.1412/0.7071	1.014 [0.609–1.688]	0.0027/0.9588
Drinking status		3.3601/0.1864 for trend		2.9514/0.2286 for trend
Never (ref 1.00)				
Previous	1.695 [0.449–6.391]	0.6063/0.4362	1.717 [0.404–7.294]	0.5370/0.4637
Current	1.384 [0.964–1.988]	3.1085/0.0779	1.311 [0.880–1.953]	1.7728/0.1830
Comorbidity				
CRS				
Yes	0.868 [0.550–1.371]	0.3675/0.5444	0.909 [0.556–1.487]	0.1444/0.7040
No (ref 1.00)				
Asthma				
Yes	0.844 [0.364–1.953]	0.1577/0.6913	0.936 [0.369–2.376]	0.0191/0.8900
No (ref 1.00)				
Bronchitis				
Yes	1.010 [0.501–2.036]	0.0007/0.9787	1.132 [0.523–2.450]	0.0990/0.7531
No (ref 1.00)				
Allergic conjunctivitis				
Yes	0.565 [0.334–0.956]	4.5307/0.0333*	0.525 [0.296–0.929]	4.8893/0.0270*
No (ref 1.0)				
Skin allergy				
Yes	1.264 [0.843–1.895]	1.2856/0.2569	1.306 [0.838–2.036]	1.3950/0.2376
No (ref 1.0)				
Food allergy				
Yes	5.858 [1.395–24.598]	5.8306/0.0157*	6.404 [1.349–30.402]	5.4604/0.0195*
No (ref 1.0)				
Vaccination frequency		25.2623/<0.0001*** for trend		25.5406/<0.0001*** for trend
0 (ref. 1.0)				
1	0.429 [0.148–1.245]	2.4245/0.1195	0.446 [0.146–1.362]	2.0117/0.1561
2	1.145 [0.485–2.704]	0.0950/0.7579	1.254 [0.516–3.048]	0.2502/0.6170
3	1.108 [0.507–2.420]	0.0662/0.7969	1.124 [0.502–2.516]	0.0812/0.7757
4	0.106 [0.030–0.376]	12.0831/0.0005***	0.093 [0.025–0.348]	12.4638/0.0004***
Previous SARS-CoV-2 infection		3.2695/0.3519		1.7968/0.6156
0 (ref. 1.0)				
1	0.545 [0.282–1.052]	3.2691/0.0706	0.616 [0.304–1.251]	1.7965/0.1801
2	>999.999 [<0.001 ->999.999]	0.0002/0.9874	>999.999 [<0.001 ->999.999]	0.0002/0.9876
≥3	>999.999 [<0.001 - >999.999]	0.0001/0.9925	>999.999 [<0.001->999.999]	0.0001/0.9923
Medication for AR or complication				
Yes	1.054 [0.767–1.449]	0.1054/0.7454	1.062 [0.751–1.502]	0.1167/0.7327
No (ref. 1.0)				

^a^ Adjusted for age, sex, weight, height, BMI, region of residence, education, occupation, smoking and drinking, comorbidity, medication, vaccination and previous infection.

Numbers in boldface indicate significant differences (*P* < 0.05). *P < 0.05, **P < 0.01, ***P < 0.001.

AR comorbid without AC was used as the reference, and AR comorbid with AC was a protective factor against SARS-CoV-2 infection both after sex and age adjustment and fully multivariable adjustment conditions (Figure 2 and Table 2). AR not comorbid without food allergy was used as the reference, and AR comorbid with food allergy was a risk factor for SARS-CoV-2 infection. The risk fold was 5.858 (95% CI = 1.395–24.598, *P* = 0.0157) and 6.404 (95% CI = 1.349–30.402, *P* = 0.0195) under the two methods of adjustment, respectively (Figure 2 and Table 2). In addition, vaccination significantly influenced the prevalence of COVID-19. Fewer patients received four doses of the vaccine, which was a significant protective factor against SARS-CoV-2 infection in AR cohort patients under both methods of adjustment [OR: 0.106 (95% CI = 0.030–0.376), *P* = 0.0005; OR: 0.093 (95% CI = 0.025–0.348), *P* = 0.0004, respectively] (Figure 2 and Table 2).

We also analyzed the factors influencing the AR cohort under the stricter definition of COVID-19. AR comorbid with food allergy is still a risk factor, and receiving four doses of the vaccine has protective effects against COVID-19. In addition, age, weight, BMI, residence in urban areas, and occupation in healthcare may be risk factors for SARS-CoV-2 infection (Supplementary Table S1 and Figure S1).

### Effect of BMI, age, and allergy-specific AR on COVID-19

**Figure 3:**
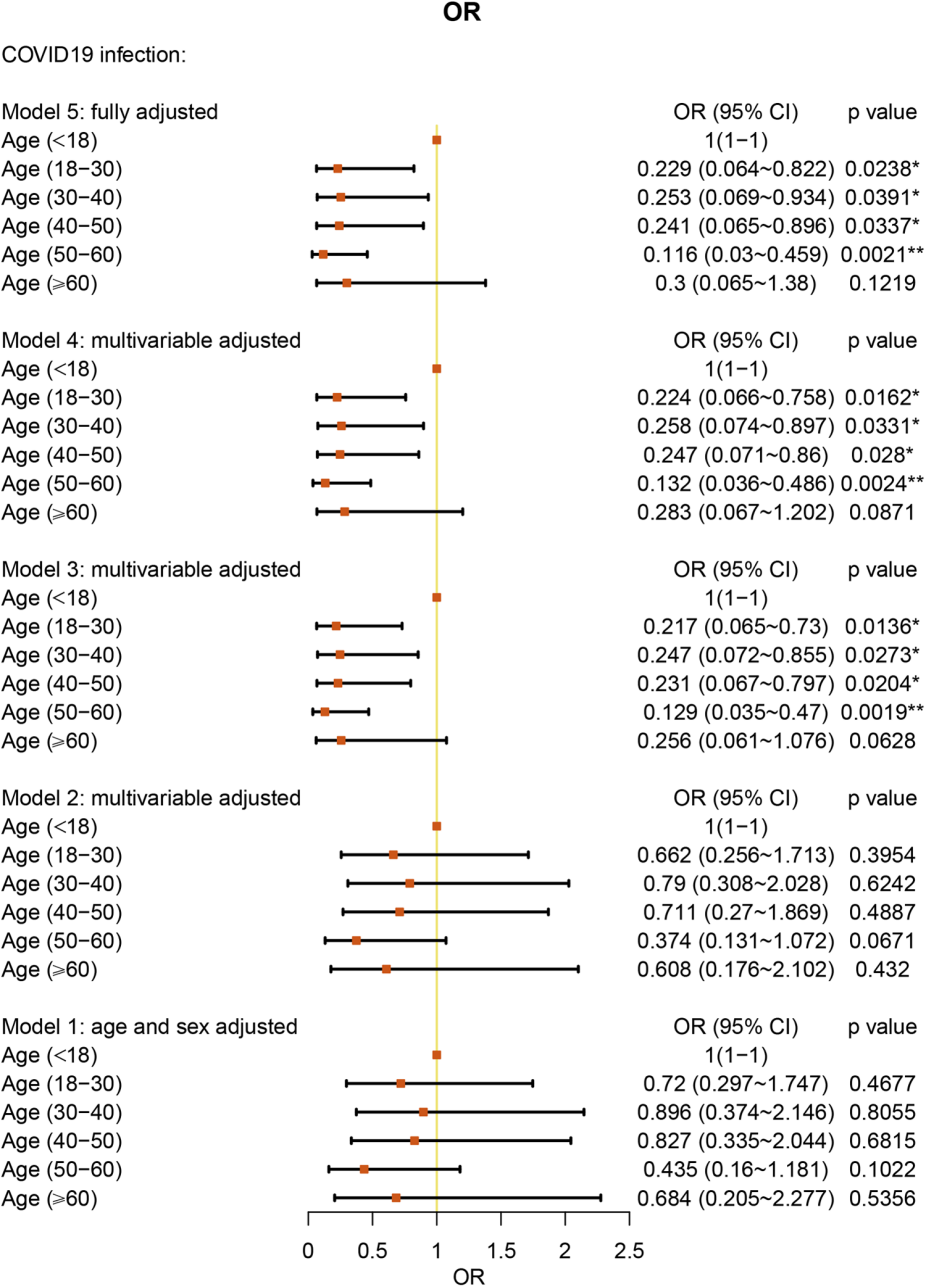
Age stratification ORs for COVID-19 in AR patients.

We analyzed the age of the patients by categories: younger than 18 years old, 18–30 years old, 30–40 years old, 40–50 years old, 50–60 years old, and older than 60 years old. With age younger than 18 years old as the reference, under the fully multivariable adjusted model, age under 60 years old showed a significant protective factor against COVID-19 in AR patients (all *P* values < 0.05) (Figure 3 and Supplementary Table S2).

**Figure 4:**
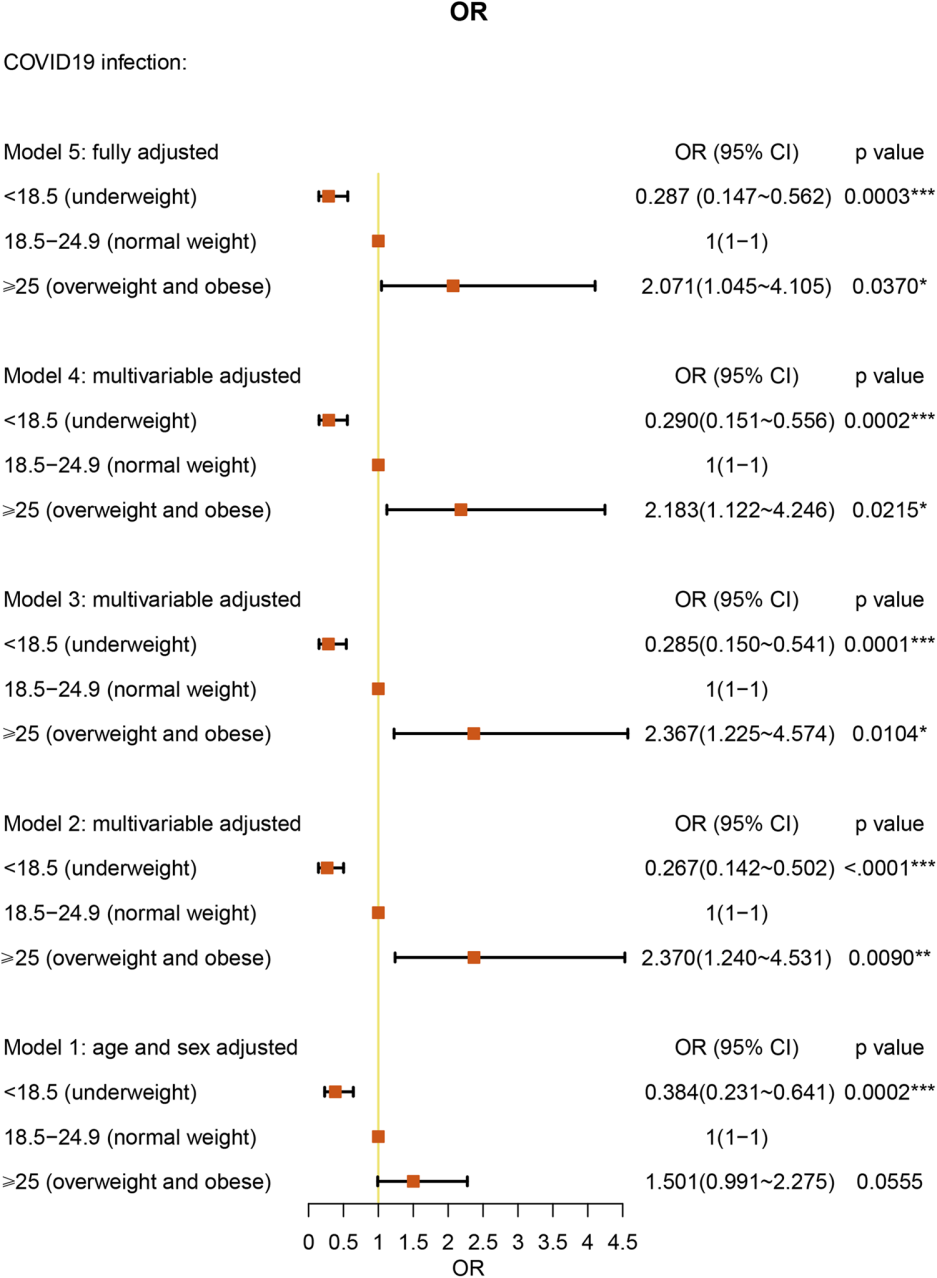
BMI stratification ORs for COVID-19 in AR patients.

We analyzed the BMI of the patients by categories: underweight (BMI < 18.5 kg/m^2^), normal weight (BMI = 18.5–24.9 kg/m^2^), and overweight (BMI > 25.0 kg/m^2^). Interestingly, underweight BMI had a protective effect against COVID-19 [OR: 0.287 (95% CI = 0.147–0.562), *P* = 0.0003] under full multivariable adjustment (model 5). Overweight BMI was associated with a 2.071-fold higher risk for COVID-19 compared with normal weight AR patients [(95% CI = 1.045–4.105), *P* = 0.0370] (Figure 4 and Supplementary Table S3).

**Figure 5:**
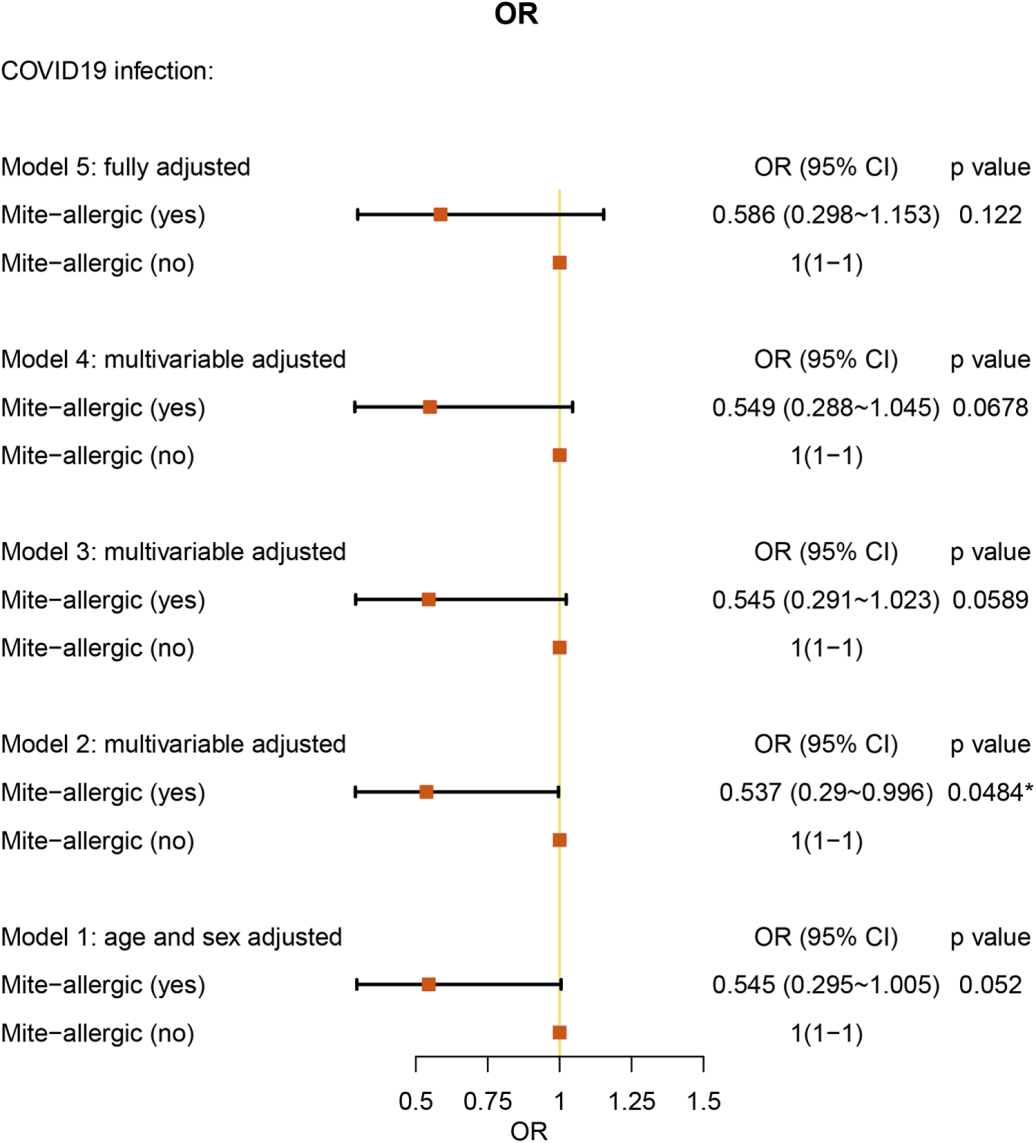
Allergen (mite)-specific multiple-adjusted ORs for COVID-19 associated with risk factors in AR patients (with allergen testing results).

We analyzed 237 AR patients with specific allergens. Only HDM-specific AR patients showed protective factors against COVID-19 [OR: 0.537 (95% CI = 0.290–0.996), *P* = 0.0484] after adjusting for age, sex, weight, and height (Figure 5 and Supplementary Table S4). For specific allergies to mites, pollen, mold, fur, or other allergens, we did not find a significant association with COVID-19 (Supplementary Table S4 and Figure S2). The geographical information, the STROBE checklist and blank questionnaire template were provided as Supplementary Figure S3, Table S5 and S6.

## Discussion

In the present nationwide retrospective cohort study, we investigated the development of COVID-19 among 830 AR patients. Furthermore, we studied the association between the clinical outcomes of COVID-19 and the characteristics of AR. The results showed complex associations between age, BMI, comorbidity with allergic diseases, type of allergen, and frequency of vaccination and COVID-19 in patients with AR.

During the study period, China experienced the peak of the epidemic after the adjustment of the national prevention and control strategy of COVID-19 infection. The present study shows that during this period, the overall infection rate (75.54%) was much higher than before (1.8% in Wuhan and Zhuhai SARS-CoV-2 infection hospitalized patients (8) and 9.8% in Wuhan COVID-19-positive patients (11)). Of note, our infection rate (75.54%) was similar to the SARS-CoV-2 infection rate of 77.89% in Beijing city (*n* = 873) (Dec. 20∼23, 2023) in the same study period (14).

In a large cohort of patients with AR, we performed a category analysis based on BMI (<18.5 kg/m^2^, 18.5–24.9 kg/m^2^, and > 25.0 kg/m^2^). Similar to the studies in all patients (15), using normal BMI as the reference, overweight BMI was associated with a 2.038-fold higher risk of COVID-19 in the AR cohort. As other studies have reported, obesity (BMI ≥ 30 kg/m^2^) was associated with a significantly increased risk of critical COVID-19 and in-hospital mortality due to COVID-19 (16, 17). In addition, we found that underweight BMI was a protective factor against COVID-19 in AR patients. However, one study reported that underweight patients were more likely than normal-weight patients to develop secondary infections (18). However, the number of individuals in this study was only 11 patients and is not large enough to explain the relationship between underweight and COVID-19. Further research is warranted to clarify this interesting association. We also found a correlation between age and COVID-19. Under fully multivariable adjusted conditions, age under 60 years old was a protective factor against COVID-19 in AR patients. The result was similar to the advocation that age under 65 years old acts as a protective factor in asthma patients (12). Our study did not present old age as a significant risk factor for COVID-19. However, one study has already shown that older patients have a higher percentage of hospitalizations, ICU admissions, and deaths (19).

Comorbidities, including asthma, atopic dermatitis, allergic rhinitis, allergic conjunctivitis, chronic rhinosinusitis, and food allergy, represent a collection of disorders mediated by innate and adaptive immune responses. After evaluating 830 patients for AR comorbid with other types of allergic diseases and prevalence of COVID-19 positivity, our results showed that AR comorbid with AC was a major protective factor against COVID-19 and that AR comorbid with food allergy was a major risk factor for COVID-19 under the fully multivariable adjusted condition. In our cohort, 5.3% of patients with AR comorbid with AC were COVID-19 positive, which was lower than 10.8% in an Italian study (20). We consider the reason why AC has a protective effect. According to previous results, thirty-eight genes involved in the defense response to virus were overexpressed, while ACE II had low expression in conjunctivitis (21). In addition, the typical Th2 phenotype and eosinophilia may be important protective factors against COVID-19. Although there is no direct explanation for the protective effect of AR comorbid with AC against COVID-19, AR patients have a strengthened type 2 immune response to some extent. This may explain our results. The main clinical symptoms of food allergy are impaired skin barrier or gastrointestinal tract accompanied by basophil and mast cell activation. Seibold et al. found food allergies associated with lower risk of COVID-19 infection (22). In asthma cohort, there were more food allergy comorbid in COVID-19 infection than non-COVID-19 infection patients by Francisco's study (23). This study was similar to our study, food allergy comorbid with AR was a risk factor for COVID-19 infection. We consider that both food allergy and AR are IgE-mediated immune responses. Patients with AR comorbid with food allergy are in a state of prolonged B-cell activation. In addition, SARS-CoV-2 infection elicits an IgE response, and the serum levels positively correlate with the severity of COVID-19 (24). Therefore, we suggest that the immune responses resulting from B cell activation could explain why AR comorbid with food allergy is a risk factor for COVID-19.

During the period of our study, the predominant variant of SARS-CoV-2 was omicron. According to previous study, two doses of vaccine only offered limited protection against the omicron variant, and the third dose could be effective in preventing patients' symptoms and severe disease (25). More importantly, a fourth dose is more effective in preventing COVID-19 compared with three standard doses of vaccine (26). In our study, it was also clear that receiving four doses of the vaccine had a significant protective effect in patients with AR, while three doses did not show protection against COVID-19 in AR patients (cohort with large sample size verification is required). In addition, even after full multivariable adjustment, four doses showed a protective factor.

House dust mite (HDM)-specific AR is prevalent due to the ubiquitous and perennial presence of HDMs in indoor environments worldwide (27). From an immune characteristic perspective, HDM-allergic AR patients show increased group 2 innate lymphoid cells (ILC-2s) and high levels of secretion of IL-5 and IL-13 (28). In COVID-19 immunopathology, the interferon (IFN) response results in exacerbated proinflammatory cytokine production in the respiratory tract and lung pathology (29). In the nasal tissue and epithelial cells of COVID-19 patients, type 2 cytokines are downregulated, while IFNs are unregulated. In addition, allergen stimulation of lower airways induces a reduction in ACE II expression, suggesting that allergic inflammation may play a significant role in lowering the risk of COVID-19. In addition, patients with HDM-specific AR are permanently exposed to an immune environment mediated by type 2 inflammation response (30). This may explain why HDM-specific AR was found to be a protective factor in COVID-19 patients.

There existed some limitations in our study. First, our study was not a clinical study, and all patients included in the study provided information by filling in online questionnaires using mobile phones. Second, some history of comorbidity data in our study was taken from the patient's self-report only. Third, we only recorded the frequency of vaccinations given to participants, without carefully distinguishing the type of vaccines. Fourth, the subjects of our study were all Chinese, and there was a lack of inclusion of other ethnic groups. Although this is a nationwide survey, the sample size is not big. Study with large sample size verification is required. Other limitations including most of the participants who were able to successfully complete the online survey were young people, with a median age of 34, which may be subject to confounding bias, etc. Despite these limitations, to our knowledge, this is the first time that a questionnaire has been used to explore risk factors in AR patients during the COVID-19 peak in China. The advantages of our cohort study are that the collection time was short, and the survey time coincided with the epidemic peak.

Of note, the SARS-CoV-2 variants at the peak of the Chinese epidemic in 2022–2023 are quite different from the main prevalent variants now. Moreover, the prevalence of SARS-CoV-2 variants varies globally, which may be one of the reasons for the conflicting results of several previous studies. Cohort study involved different countries with large sample size verification are required.

In summary, the different clinical manifestations of AR patients are closely associated with COVID-19. We analyzed the demographic characteristics of the patients, and BMI and age had a significant impact on COVID-19. Underweight BMI and age lower than 60 years were protective factors, and overweight BMI was a risk factor for COVID-19 after full multivariable adjustment. We present for the first time the effect of different allergic disease comorbidities on COVID-19 in AR patients. AR comorbid with AC and AR comorbid with food allergy have the opposite effect on COVID-19. HDM-specific AR patients, compared to other AR patients, are better protected against COVID-19.

## Data Availability

The original contributions presented in the study are included in the article/Supplementary Material, further inquiries can be directed to the corresponding authors.

## References

[1] Wu SL Mertens AN Crider YS Nguyen A Pokpongkiat NN Djajadi S Substantial underestimation of Sars-Cov-2 infection in the United States. Nat Commun. (2020) 11(1):4507. 10.1038/s41467-020-18272-4 32908126 PMC7481226

[2] Zhang Y Lan F Zhang L. Advances and highlights in allergic rhinitis. Allergy. (2021) 76(11):3383–9. 10.1111/all.15044 34379805

[3] Galli SJ Tsai M Piliponsky AM. The development of allergic inflammation. Nature. (2008) 454(7203):445–54. 10.1038/nature07204 18650915 PMC3573758

[4] Juhn YJ. Risks for infection in Patients with asthma (or other atopic conditions): is asthma more than a chronic airway disease? J Allergy Clin Immunol. (2014) 134(2):247–57, quiz 58-9. 10.1016/j.jaci.2014.04.024 25087224 PMC4122981

[5] Brożek JL Bousquet J Agache I Agarwal A Bachert C Bosnic-Anticevich S Allergic rhinitis and its impact on asthma (Aria) guidelines-2016 revision. J Allergy Clin Immunol. (2017) 140(4):950–8. 10.1016/j.jaci.2017.03.050 28602936

[6] Wang XD Zheng M Lou HF Wang CS Zhang Y Bo MY An increased prevalence of self-reported allergic rhinitis in Major Chinese cities from 2005 to 2011. Allergy. (2016) 71(8):1170–80. 10.1111/all.12874 26948849 PMC5074323

[7] Choi HG Kim SY Joo YH Cho HJ Kim SW Jeon YJ. Incidence of asthma, atopic dermatitis, and allergic rhinitis in Korean adults before and during the COVID-19 pandemic using data from the Korea National Health and nutrition examination survey. Int J Environ Res Public Health. (2022) 19(21):14274. 10.3390/ijerph192114274 36361154 PMC9658105

[8] Jian L Yi W Zhang N Wen W Krysko O Song WJ Perspective: COVID-19, Implications of nasal diseases and consequences for their management. J Allergy Clin Immunol. (2020) 146(1):67–9. 10.1016/j.jaci.2020.04.030 32360869 PMC7252138

[9] Yang JM Koh HY Moon SY Yoo IK Ha EK You S Allergic disorders and susceptibility to and severity of COVID-19: a nationwide cohort study. J Allergy Clin Immunol. (2020) 146(4):790–8. 10.1016/j.jaci.2020.08.008 32810517 PMC7428784

[10] Yang X Zhao J Sima Y Zhao Y Zhang J Wang X The association between allergic rhinitis and the risk of coronavirus disease 2019 (COVID-19): a national survey in China. Allergy. (2023) 78(10):2783–6. 10.1111/all.15823 37476953

[11] Wang H Song J Yao Y Deng YK Wang ZC Liao B Angiotensin-Converting enzyme ii expression and its implication in the association between COVID-19 and allergic rhinitis. Allergy. (2021) 76(3):906–10. 10.1111/all.14569 32851645 PMC7461276

[12] Ren J Pang W Luo Y Cheng D Qiu K Rao Y Impact of allergic rhinitis and asthma on COVID-19 infection, hospitalization, and mortality. J Allergy Clin Immunol Pract. (2022) 10(1):124–33. 10.1016/j.jaip.2021.10.049 34728408 PMC8556867

[13] Darabi A Dehghanfard M Jozan S Tahmasebi R Movahed A Zamani M Investigating the association between allergic diseases and COVID-19 in 400 Iranian patients. Allergol Immunopathol (Madr). (2021) 49(5):9–15. 10.15586/aei.v49i5.105 34476916

[14] Wei YY Gao WJ Zhang LY Wang SG Zhang SY Ren T Epidemiological survey of 2019-nCoV infection in staff and students in some public health schools in China. Chin J Epidemiol. (2023) 44(2):175–83. 10.3760/cma.j.cn112338-20221231-01092

[15] Du Y Lv Y Zha W Zhou N Hong X. Association of body mass Index (bmi) with critical COVID-19 and in-hospital mortality: a dose-response meta-analysis. Metab Clin Exp. (2021) 117:154373. 10.1016/j.metabol.2020.154373 32949592 PMC7493748

[16] Hendren NS de Lemos JA Ayers C Das SR Rao A Carter S Association of body mass Index and age with morbidity and mortality in patients hospitalized with COVID-19: results from the American Heart Association COVID-19 cardiovascular disease registry. Circulation. (2021) 143(2):135–44. 10.1161/CIRCULATIONAHA.120.051936 33200947

[17] Sanchis-Gomar F Lavie CJ Mehra MR Henry BM Lippi G. Obesity and outcomes in COVID-19: when an epidemic and pandemic collide. Mayo Clin Proc. (2020) 95(7):1445–53. 10.1016/j.mayocp.2020.05.006 32622449 PMC7236707

[18] Ye P Pang R Li L Li HR Liu SL Zhao L. Both underweight and obesity are associated with an increased risk of coronavirus disease 2019 (COVID-19) severity. Front Nutr. (2021) 8:649422. 10.3389/fnut.2021.649422 34692741 PMC8531071

[19] Chen Y Klein SL Garibaldi BT Li H Wu C Osevala NM Aging in COVID-19: vulnerability, immunity and intervention. Ageing Res Rev. (2021) 65:101205. 10.1016/j.arr.2020.101205 33137510 PMC7604159

[20] Ferreli F Gaino F Russo E Di Bari M Pirola F Costantino A Clinical presentation at the onset of COVID-19 and allergic rhinoconjunctivitis. J Allergy Clin Immunol Pract. (2020) 8(10):3587–9. 10.1016/j.jaip.2020.08.009 32818700 PMC7431330

[21] Leonardi A Rosani U Cavarzeran F Daull P Garrigue JS Paola B. Antiviral response in Vernal Keratoconjunctivitis may be protective against COVID-19. Allergy. (2022) 77(1):298–300. 10.1111/all.15048 34390598 PMC8441822

[22] Seibold MA Moore CM Everman JL Williams BJM Nolin JD Fairbanks-Mahnke A Risk factors for Sars-Cov-2 infection and transmission in households with children with asthma and allergy: a prospective surveillance study. J Allergy Clin Immunol. (2022) 150(2):302–11. 10.1016/j.jaci.2022.05.014 35660376 PMC9155183

[23] Ruano FJ Somoza Álvarez ML Haroun-Díaz E de la Torre M V González P L Prieto-Moreno A Impact of the COVID-19 pandemic in children with allergic asthma. J Allergy Clin Immunol Pract. (2020) 8(9):3172–4.e1. 10.1016/j.jaip.2020.07.019 32730834 PMC7384405

[24] Plūme J Galvanovskis A Šmite S Romanchikova N Zayakin P Linē A. Early and strong antibody responses to Sars-Cov-2 predict disease severity in COVID-19 patients. J Transl Med. (2022) 20(1):176. 10.1186/s12967-022-03382-y 35428263 PMC9012069

[25] Magen O Waxman JG Makov-Assif M Vered R Dicker D Hernán MA Fourth dose of Bnt162b2 Mrna COVID-19 vaccine in a nationwide setting. N Engl J Med. (2022) 386(17):1603–14. 10.1056/NEJMoa2201688 35417631 PMC9020581

[26] Regev-Yochay G Gonen T Gilboa M Mandelboim M Indenbaum V Amit S Efficacy of a fourth dose of COVID-19 Mrna vaccine against Omicron. N Engl J Med. (2022) 386(14):1377–80. 10.1056/NEJMc2202542 35297591 PMC9006792

[27] Zock JP Heinrich J Jarvis D Verlato G Norbäck D Plana E Distribution and determinants of house dust Mite allergens in Europe: The European Community Respiratory Health Survey II. J Allergy Clin Immunol. (2006) 118(3):682–90. 10.1016/j.jaci.2006.04.060 16950288

[28] Zhong H Fan XL Yu QN Qin ZL Chen D Xu R Increased innate type 2 immune response in house dust mite-allergic patients with allergic rhinitis. Clin Immunol. (2017) 183:293–9. 10.1016/j.clim.2017.09.008 28917723

[29] Lowery SA Sariol A Perlman S. Innate immune and inflammatory responses to Sars-Cov-2: implications for COVID-19. Cell Host Microbe. (2021) 29(7):1052–62. 10.1016/j.chom.2021.05.004 34022154 PMC8126603

[30] Jackson DJ Busse WW Bacharier LB Kattan M O'Connor GT Wood RA Association of respiratory allergy, asthma, and expression of the Sars-Cov-2 receptor Ace2. J Allergy Clin Immunol. (2020) 146(1):203–6.e3. 10.1016/j.jaci.2020.04.009 32333915 PMC7175851

