# Supplementary figures and images for: Risk and protective factors for coronavirus disease 2019 (COVID-19) in allergic rhinitis patients: a national survey in China
Source: Front Allergy. 2024 Dec 10;5:1479493. doi: 10.3389/falgy.2024.1479493 (PMC11666529; doi:10.3389/falgy.2024.1479493)

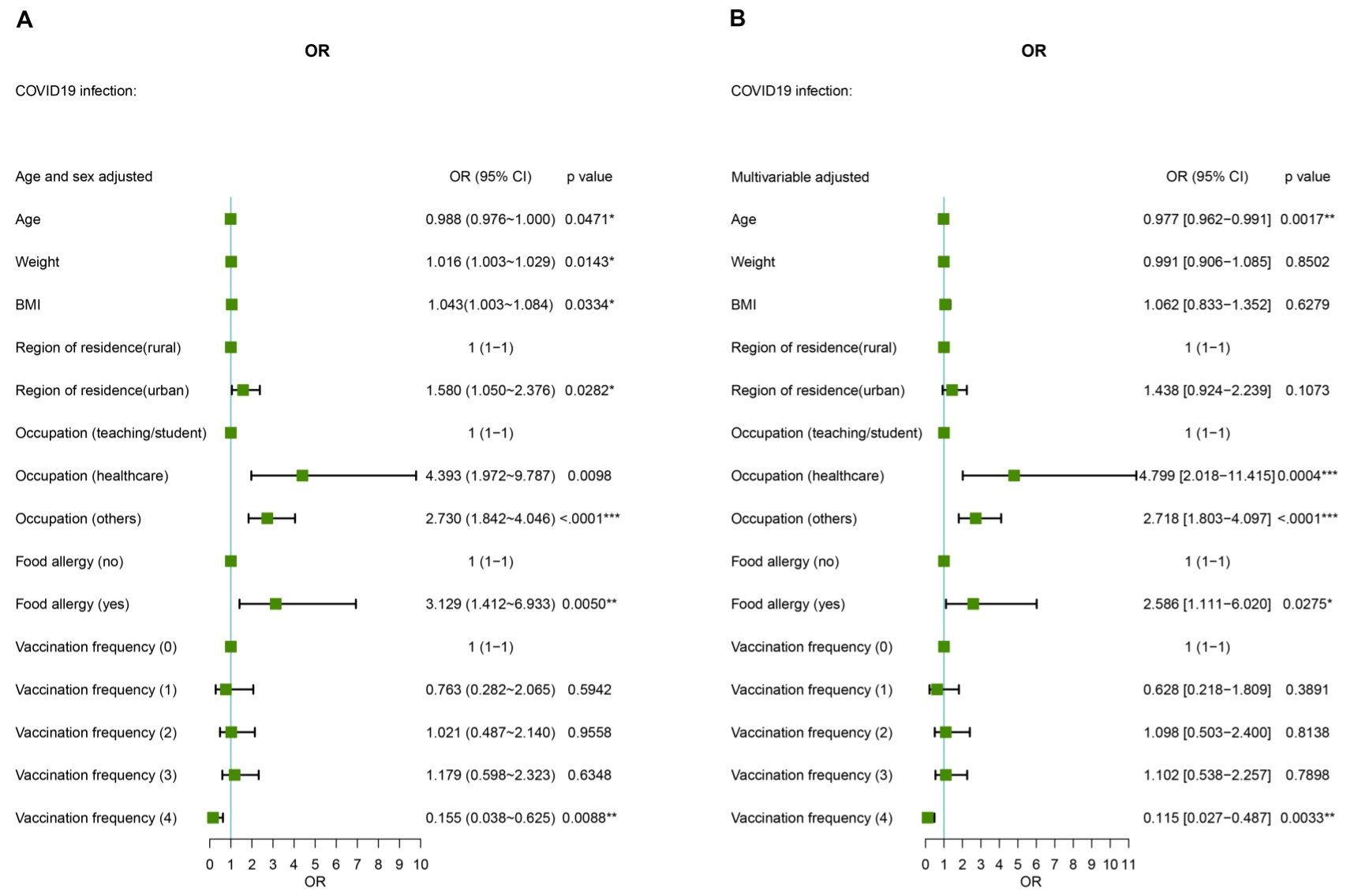

Supplement: Supplementary file 1 [file Image1.jpeg]

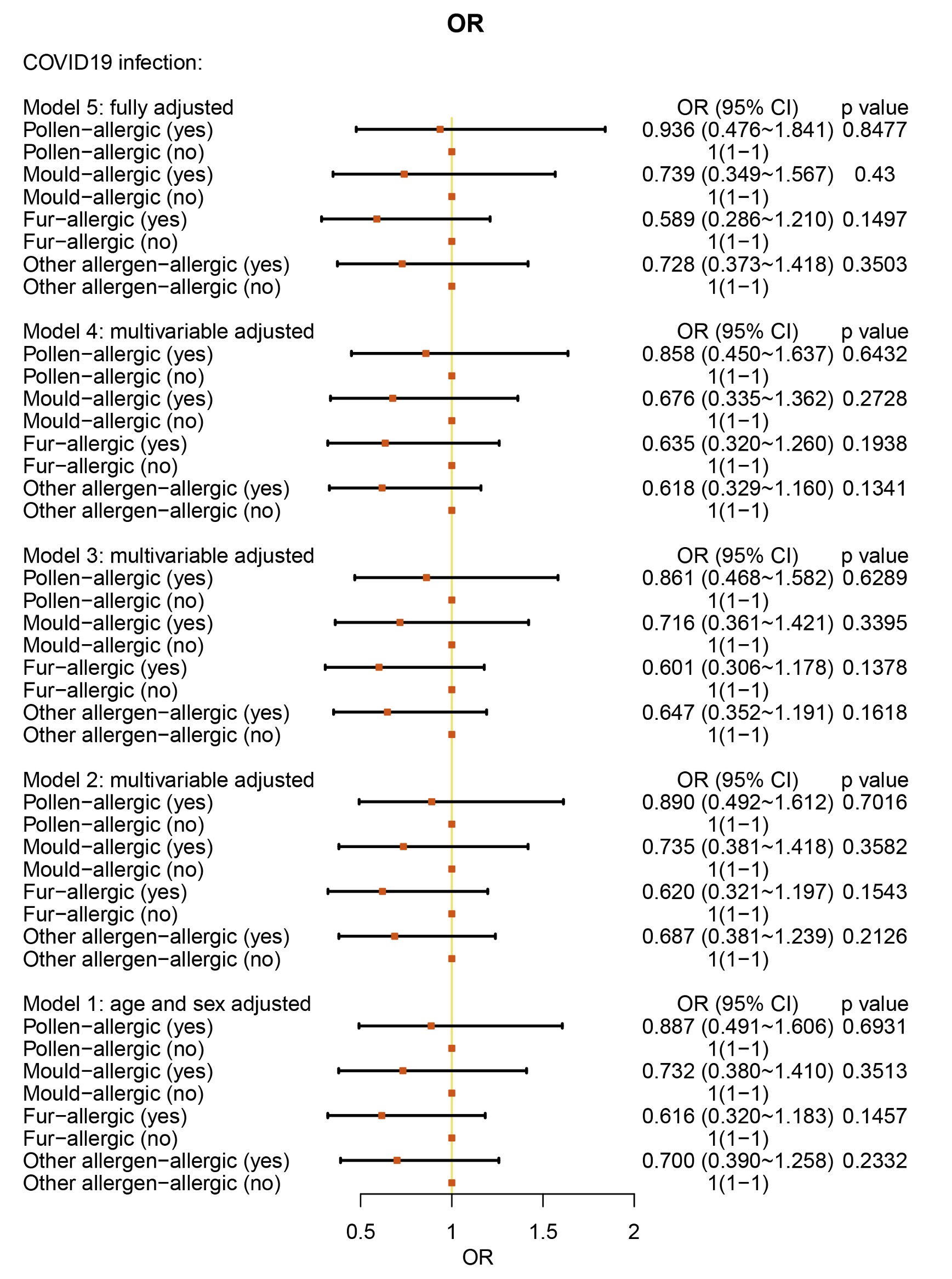

Supplement: Supplementary file 2 [file Image2.jpeg]

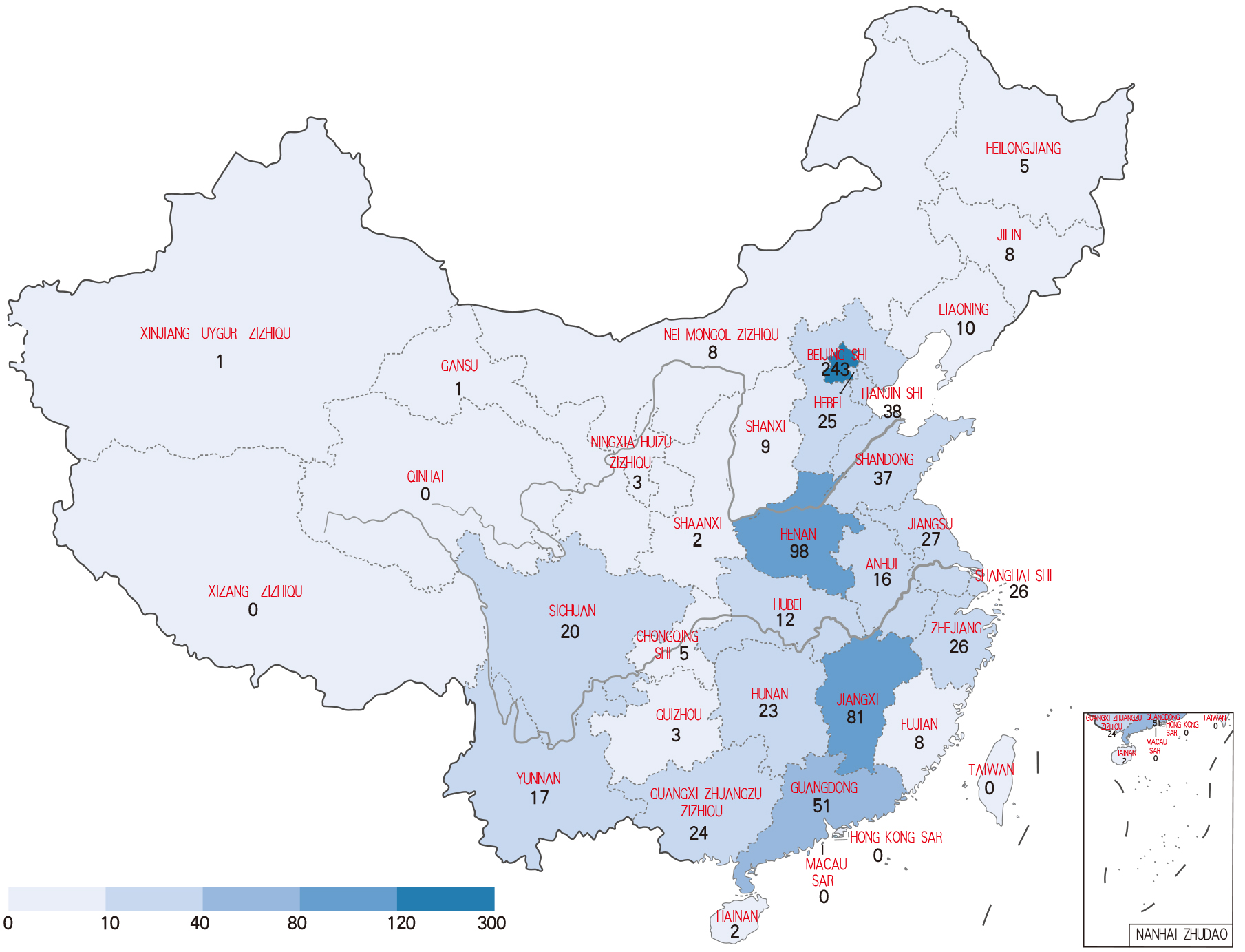

Supplement: Supplementary file 3 [file Image3.jpeg]
