# Supplementary material for: Risk and protective factors for coronavirus disease 2019 (COVID-19) in allergic rhinitis patients: a national survey in China
Source: Front Allergy. 2024 Dec 10;5:1479493. doi: 10.3389/falgy.2024.1479493 (PMC11666529; doi:10.3389/falgy.2024.1479493)
Supplement: Supplementary file 4 [file Datasheet1.docx]

Supplementary Material

**Table S1. Multiple-adjusted ORs for COVID19 infection (with PCR test positive or antigen detection positive or both of two tests are positive) associated with risk factors in AR patients (with 460 cases infected and 370 cased not infected).**

| **Characteristics** |  | **Age, sex adjusted** | |  | | **Multivariable adjusted*** | | |  |
| --- | --- | --- | --- | --- | --- | --- | --- | --- | --- |
|  |  | **OR [95%CI]** | **Waldχ^2^/P value** | |  | | **OR [95%CI]** | **Wald χ^2^/P value** | |
| **Age** |  | 0.988 [0.976- 1.000] | **3.9433/0.0471*** | |  | | 0.977 [0.962-0.991] | **9.8853/0.0017**** | |
| **Sex** |  |  |  | |  | |  |  | |
| Male |  | 1.056 [0.800- 1.393] | 0.1457/0.7026 | |  | | 0.889 [0.566-1.395] | 0.2632/0.6079 | |
| Female (ref. 1.0) |  |  |  | |  | |  |  | |
| **Height** |  | 1.011 [0.994-1.030] | 1.5684/0.2104 | |  | | 1.012 [0.944-1.084] | 0.1101/0.7400 | |
| **Weight** |  | 1.016 [1.003- 1.029] | **5.9950/0.0143*** | |  | | 0.991 [0.906-1.085] | 0.0357/0.8502 | |
| **BMI** |  | 1.043 [1.003 -1.084] | **4.5248/0.0334*** | |  | | 1.062 [0.833-1.352] | 0.2349/0.6279 | |
| **Region of residence** |  |  |  | |  | |  |  | |
| Urban |  | 1.580 [1.050- 2.376] | **4.8184/0.0282*** | |  | | 1.438 [0.924-2.239] | 2.5940/0.1073 | |
| Rural (ref. 1.0) |  |  |  | |  | |  |  | |
| **Education attainment** |  |  | **19.5341/<.0001** for trend | |  | |  | **12.7152/0.0017** for trend | |
| Primary school and lower (ref. 1.00) |  |  |  | |  | |  |  | |
| Middle and high school |  | 0.678 [0.271-1.694] | 0.6931/0.4051 | |  | | 0.431 [0.135-1.381] | 2.0051/0.1568 | |
| College and higher |  | 1.583 [0.671-3.735] | 1.0999 /0.2943 | |  | | 0.907 [0.293-2.806] | 0.0284/0.8661 | |
| **Occupation** |  |  | **27.9366/<.0001** for trend | |  | |  | **25.9464/<.0001** for trend | |
| Teaching/student (ref. 1.0) |  |  |  | |  | |  |  | |
| Healthcare |  | 4.393 [1.972-9.787] | **6.6771/0.0098** | |  | | 4.799 [2.018-11.415] | **12.5880/0.0004***** | |
| Others |  | 2.730 [1.842-4.046] | **25.02/<.0001**** | |  | | 2.718 [1.803-4.097] | **22.8092/<.0001***** | |
| **Smoking status** |  |  | 0.7260/0.6956  for trend | |  | |  | 1.3054/0.5206 for trend | |
| Never (ref. 1.0) |  |  |  | |  | |  |  | |
| Previous |  | 0.867 [0.443-1.699] | 0.1721/0.6783 | |  | | 0.708 [0.345-1.453] | 0.8854/0.3467 | |
| Current |  | 1.145 [0.770-1.703] | 0.4473/0.5036 | |  | | 1.109 [0.709-1.735] | 0.2049/0.6508 | |
| **Drinking status** |  |  | 2.4595/0.2924 for trend | |  | |  | 0.1754/0.9160 for trend | |
| Never (ref. 1.00) |  |  |  | |  | |  |  | |
| Previous |  | 1.002 [0.336-2.989] | 0.0000/0.9975 | |  | | 1.025 [0.305-3.447] | 0.0015/0.9688 | |
| Current |  | 1.276 [0.937-1.739] | 2.3922/0.1219 | |  | | 1.076 [0.762-1.519] | 0.1726/0.6778 | |
| **Comorbidity** |  |  |  | |  | |  |  | |
| **CRS** |  |  |  | |  | |  |  | |
| Yes |  | 0.879 [0.586-1.319] | 0.3893/0.5327 | |  | | 0.856[0.552-1.328] | 0.4796/0.4886 | |
| No (ref. 1.00) |  |  |  | |  | |  |  | |
| **Asthma** |  |  |  | |  | |  |  | |
| Yes |  | 1.112 [0.517-2.392] | 0.0739/0.7857 | |  | | 1.153[0.493-2.697] | 0.1083/0.7421 | |
| No (ref. 1.00) |  |  |  | |  | |  |  | |
| **Bronchitis** |  |  |  | |  | |  |  | |
| Yes |  | 0.941 [0.514-1.723] | 0.0389/0.8437 | |  | | 1.141[0.584-2.231] | 0.1489/0.6996 | |
| No (ref. 1.00) |  |  |  | |  | |  |  | |
| **Allergic conjunctivitis** |  |  |  | |  | |  |  | |
| Yes |  | 0.863 [0.524-1.420] | 0.3368/0.5617 | |  | | 0.723[0.421-1.241] | 1.3856/0.2391 | |
| No (ref. 1.0) |  |  |  | |  | |  |  | |
| **Skin allergy** |  |  |  | |  | |  |  | |
| Yes |  | 1.380 [0.980- 1.944] | 3.4071/ 0.0649 | |  | | 1.328 [0.912-1.935] | 2.1887/0.1390 | |
| No (ref. 1.00) |  |  |  | |  | |  |  | |
| **Food allergy** |  |  |  | |  | |  |  | |
| Yes |  | 3.129 [1.412- 6.933] | **7.8935/0.0050**** | |  | | 2.586 [1.111-6.020] | **4.8610/0.0275*** | |
| No (ref. 1.0) |  |  |  | |  | |  |  | |
| **Vaccination frequency** |  |  | **11.6587/0.0201*** for trend | |  | |  | **13.7556/0.0081**** for trend | |
| 0 (ref. 1.0) |  |  |  | |  | |  |  | |
| 1 |  | 0.763 [0.282- 2.065] | 0.2839/0.5942 | |  | | 0.628 [0.218-1.809] | 0.7418/0.3891 | |
| 2 |  | 1.021 [0.487- 2.140] | 0.0031/0.9558 | |  | | 1.098 [0.503-2.400] | 0.0554/0.8138 | |
| 3 |  | 1.179 [0.598- 2.323] | 0.2256/0.6348 | |  | | 1.102 [0.538-2.257] | 0.0710/0.7898 | |
| 4 |  | 0.155 [0.038- 0.625] | **6.8687/0.0088**** | |  | | 0.115 [0.027-0.487] | **8.6163/0.0033**** | |
| **Previous infection with COVID-19** | | | 0.8704/0.8326 | |  | |  | 0.3209/0.9560 | |
| 0 (ref. 1.0) |  |  |  | |  | |  |  | |
| 1 |  | 0.742 [0.397-1.389] | 0.8699/0.3510 | |  | | 0.822 [0.417-1.620] | 0.3204/0.5714 | |
| 2 |  | >999.999 [<0.001->999.999] | 0.0004/0.9842 | |  | | >999.999 [<0.001->999.999] | 0.0004/0.9836 | |
| ≥3 |  | <0.001 [<0.001->999.999] | 0.0001/0.9908 | |  | | <0.001 [<0.001->999.999] | 0.0001/0.9914 | |
| **Medication for AR or** comorbidities | | | | | | | | | |
| Yes |  | 1.210 [0.918-1.594] | 1.8355/0.1755 | |  | | 1.156[0.854-1.564] | 0.8788/0.3485 | |
| No (ref. 1.0) |  |  |  | |  | |  |  | |

*Adjustment for age, sex, weight, height, BMI, region of residence, education, occupation, smoking and drinking, comorbidity, medication, vaccination and previous infection.

**Table S2. Age stratification (n=830) ORs for COVID19 infection in AR patients.**

| **Age (years)**  **stratification** | **OR^5^** | **95% CI_low** | **95% CI_up** | **Wald χ2** | **P value** |
| --- | --- | --- | --- | --- | --- |
| **Age＜18 (ref. )** | 1.0 |  |  | 11.6280 | **0.0403*** for trend |
| **Age (18-30)** | 0.229 | 0.064 | 0.822 | 5.1063 | **0.0238*** |
| **Age (30-40)** | 0.253 | 0.069 | 0.934 | 4.2563 | **0.0391*** |
| **Age (40-50)** | 0.241 | 0.065 | 0.896 | 4.5104 | **0.0337*** |
| **Age (50-60)** | 0.116 | 0.03 | 0.459 | 9.4395 | **0.0021**** |
| **Age (≥60)** | 0.3 | 0.065 | 1.38 | 2.3927 | 0.1219 |
|  | **OR^4^** | **95% CI_low** | **95% CI_up** | **Wald χ2** | **P value** |
| **Age＜18 (ref. )** | 1.0 |  |  | 10.9037 | 0.0533 for trend |
| **Age (18-30)** | 0.224 | 0.066 | 0.758 | 5.7862 | **0.0162*** |
| **Age (30-40)** | 0.258 | 0.074 | 0.897 | 4.5385 | **0.0331*** |
| **Age (40-50)** | 0.247 | 0.071 | 0.86 | 4.828 | **0.028*** |
| **Age (50-60)** | 0.132 | 0.036 | 0.486 | 9.2529 | **0.0024**** |
| **Age (≥60)** | 0.283 | 0.067 | 1.202 | 2.9267 | 0.0871 |
|  | **OR^3^** | **95% CI_low** | **95% CI_up** | **Wald χ2** | **P value** |
| **Age＜18 (ref.)** | 1.0 |  |  | 11.0815 | **0.0498*** for trend |
| **Age (18-30)** | 0.217 | 0.065 | 0.73 | 6.0957 | **0.0136*** |
| **Age (30-40)** | 0.247 | 0.072 | 0.855 | 4.8715 | **0.0273*** |
| **Age (40-50)** | 0.231 | 0.067 | 0.797 | 5.3783 | **0.0204*** |
| **Age (50-60)** | 0.129 | 0.035 | 0.47 | 9.618 | **0.0019**** |
| **Age (≥60)** | 0.256 | 0.061 | 1.076 | 3.4606 | 0.0628 |
|  | **OR^2^** | **95% CI_low** | **95% CI_up** | **Wald χ2** | **P value** |
| **Age＜18 (ref.)** | 1.0 |  |  | 6.5895 | 0.2530 for trend |
| **Age (18-30)** | 0.662 | 0.256 | 1.713 | 0.7222 | 0.3954 |
| **Age (30-40)** | 0.79 | 0.308 | 2.028 | 0.2399 | 0.6242 |
| **Age (40-50)** | 0.711 | 0.27 | 1.869 | 0.4793 | 0.4887 |
| **Age (50-60)** | 0.374 | 0.131 | 1.072 | 3.3526 | 0.0671 |
| **Age (≥60)** | 0.608 | 0.176 | 2.102 | 0.6174 | 0.432 |
|  | **OR^1^** | **95% CI_low** | **95% CI_up** | **Wald χ2** | **P value** |
| **Age＜18 (ref. )** | 1.0 |  |  | 6.1674 | 0.2903 for trend |
| **Age (18-30)** | 0.72 | 0.297 | 1.747 | 0.5275 | 0.4677 |
| **Age (30-40)** | 0.896 | 0.374 | 2.146 | 0.0606 | 0.8055 |
| **Age (40-50)** | 0.827 | 0.335 | 2.044 | 0.1684 | 0.6815 |
| **Age (50-60)** | 0.435 | 0.16 | 1.181 | 2.6702 | 0.1022 |
| **Age (≥60)** | 0.684 | 0.205 | 2.277 | 0.3838 | 0.5356 |

Ref., reference

1. Adjustment for age, sex.

2. Adjustment for age, sex, weight, height.

3. Adjustment for age, sex, weight, height, BMI, region of residence, education, occupation, smoking and drinking.

4. Adjustment for age, sex, weight, height, BMI, region of residence, education, occupation, smoking and drinking, comorbidity, medication.

5. Adjustment for age, sex, weight, height, BMI, region of residence, education, occupation, smoking and drinking, comorbidity, medication, vaccination and previous infection.

**Table S3. BMI stratification (n=830) ORs for COVID19 infection in AR patients**

| **BMI stratification** | **OR^5^** | **CI_low** | **CI_up** | **Wald χ2** | **P value** |
| --- | --- | --- | --- | --- | --- |
|  |  |  |  | 5.6250 | 0.0601 for trend |
| **<18.5 (underweight)** | 0.287 | 0.147 | 0.562 | 13.2906 | 0.0003*** |
| **18.5-24.9 (normal weight)** | ref. 1.0 |  |  |  |  |
| **≥25 (overweight and obese)** | 2.071 | 1.045 | 4.105 | 4.3524 | 0.0370* |
|  | **OR^4^** | **CI_low** | **CI_up** | **Wald χ2** | **P value** |
|  |  |  |  | 5.7214 | 0.0572 for trend |
| **<18.5 (underweight)** | 0.290 | 0.151 | 0.556 | 13.8891 | 0.0002*** |
| **18.5-24.9 (normal weight)** | ref. 1.0 |  |  |  |  |
| **≥25 (overweight and obese)** | 2.183 | 1.122 | 4.246 | 5.2850 | 0.0215* |
|  | **OR^3^** | **CI_low** | **CI_up** | **Wald χ2** | **P value** |
|  |  |  |  | 5.6128 | 0.0604 for trend |
| **<18.5 (underweight)** | 0.285 | 0.150 | 0.541 | 14.6913 | 0.0001*** |
| **18.5-24.9 (normal weight)** | ref. 1.0 |  |  |  |  |
| **≥25 (overweight and obese)** | 2.367 | 1.225 | 4.574 | 6.5694 | 0.0104* |
|  | **OR^2^** | **CI_low** | **CI_up** | **Wald χ2** | **P value** |
|  |  |  |  | 0.7023 | 0.7039 for trend |
| **<18.5 (underweight)** | 0.267 | 0.142 | 0.502 | 16.7793 | <.0001*** |
| **18.5-24.9 (normal weight)** | ref. 1.0 |  |  |  |  |
| **≥25 (overweight and obese)** | 2.370 | 1.240 | 4.531 | 6.8162 | 0.0090** |
|  | **OR^1^** | **CI_low** | **CI_up** | **Wald χ2** | **P value** |
|  |  |  |  | 0.7249 | 0.6960 for trend |
| **<18.5 (underweight)** | 0.384 | 0.231 | 0.641 | 13.4482 | 0.0002*** |
| **18.5-24.9 (normal weight)** | ref. 1.0 |  |  |  |  |
| **≥25 (overweight and obese)** | 1.501 | 0.991 | 2.275 | 3.6674 | 0.0555 |

1. Adjustment for age, sex, BMI.

2. Adjustment for age, sex, weight, height, BMI.

3. Adjustment for age, sex, weight, height, BMI, region of residence, education, occupation, smoking and drinking.

4. Adjustment for age, sex, weight, height, BMI, region of residence, education, occupation, smoking and drinking, comorbidity, treatment.

5. Adjustment for age, sex, weight, height, BMI, region of residence, education, occupation, smoking and drinking, comorbidity, treatment, vaccination and previous infection.

**Table S4. Allergen specific multiple-adjusted ORs for COVID19 infection associated with risk factors in AR patients (with allergen testing results)(n=237).**

| **Allergen test** | **OR^1^** | **95%CI_low** | **95%CI_high** | **Wald χ^2^/P value** |
| --- | --- | --- | --- | --- |
| **Mite-allergic** |  |  |  |  |
| Yes | 0.545 | 0.295 | 1.005 | 3.7769/ 0.0520 |
| No (ref. 1.0) |  |  |  |  |
| **Pollen-allergic** |  |  |  |  |
| Yes | 0.887 | 0.491 | 1.606 | 0.1558 /0.6931 |
| No (ref 1.0) |  |  |  |  |
| **Mould-allergic** |  |  |  |  |
| Yes | 0.732 | 0.380 | 1.410 | 0.8689/0.3513 |
| No (ref. 1.0) |  |  |  |  |
| **Fur allergy (cat, dog *ect.*)** |  |  |  |  |
| Yes | 0.616 | 0.320 | 1.183 | 2.1171/0.1457 |
| No (ref. 1.0) |  |  |  |  |
| **Other allergen-allergic** |  |  |  |  |
| Yes | 0.700 | 0.390 | 1.258 | 1.4213/0.2332 |
| No (ref. 1.0) |  |  |  |  |
| **Allergen test** | **OR^2^** | **95%CI_low** | **95%CI_high** | **Wald χ^2^/P value** |
| **Mite-allergic** |  |  |  |  |
| Yes | 0.537 | 0.290 | 0.996 | **3.8953/0.0484*** |
| No (ref. 1.0) |  |  |  |  |
| **Pollen-allergic** |  |  |  |  |
| Yes | 0.890 | 0.492 | 1.612 | 0.1468/0.7016 |
| No (ref. 1.0) |  |  |  |  |
| **Mould-allergic** |  |  |  |  |
| Yes | 0.735 | 0.381 | 1.418 | 0.8443/0.3582 |
| No (ref. 1.0) |  |  |  |  |
| **Fur allergy (cat, dog *ect.*)** |  |  |  |  |
| Yes | 0.620 | 0.321 | 1.197 | 2.0295/0.1543 |
| No (ref. 1.0) |  |  |  |  |
| **Other allergen-allergic** |  |  |  |  |
| Yes | 0.687 | 0.381 | 1.239 | 1.5539/0.2126 |
| No (ref. 1.0) |  |  |  |  |
| **Allergen test** | **OR^3^** | **95%CI_low** | **95%CI_high** | **Wald χ^2^/P value** |
| **Mite-allergic** |  |  |  |  |
| Yes | 0.545 | 0.291 | 1.023 | 3.5673/0.0589 |
| No (ref. 1.0) |  |  |  |  |
| **Pollen-allergic** |  |  |  |  |
| Yes | 0.861 | 0.468 | 1.582 | 0.2335/0.6289 |
| No (ref. 1.0) |  |  |  |  |
| **Mould-allergic** |  |  |  |  |
| Yes | 0.716 | 0.361 | 1.421 | 0.9123/0.3395 |
| No (ref. 1.0) |  |  |  |  |
| **Fur allergy (cat, dog *ect.*)** |  |  |  |  |
| Yes | 0.601 | 0.306 | 1.178 | 2.2029/0.1378 |
| No (ref. 1.0) |  |  |  |  |
| **Other allergen-allergic** |  |  |  |  |
| Yes | 0.647 | 0.352 | 1.191 | 1.9573/0.1618 |
| No (ref. 1.0) |  |  |  |  |
| **Allergen test** | **OR^4^** | **95%CI_low** | **95%CI_high** | **Wald χ^2^/P value** |
| **Mite-allergic** |  |  |  |  |
| Yes | 0.549 | 0.288 | 1.045 | 3.3346/0.0678 |
| No (ref. 1.0) |  |  |  |  |
| **Pollen-allergic** |  |  |  |  |
| Yes | 0.858 | 0.450 | 1.637 | 0.2146/0.6432 |
| No (ref. 1.0) |  |  |  |  |
| **Mould-allergic** |  |  |  |  |
| Yes | 0.676 | 0.335 | 1.362 | 1.2026/0.2728 |
| No (ref. 1.0) |  |  |  |  |
| **Fur allergy (cat, dog *ect.*)** |  |  |  |  |
| Yes | 0.635 | 0.320 | 1.260 | 1.6884/0.1938 |
| No (ref. 1.0) |  |  |  |  |
| **Other allergen-allergic** |  |  |  |  |
| Yes | 0.618 | 0.329 | 1.160 | 2.2446/0.1341 |
| No (ref. 1.0) |  |  |  |  |
| **Allergen test** | **OR^5^** | **95%CI_low** | **95%CI_high** | **Wald χ^2^/P value** |
| **Mite-allergic** |  |  |  |  |
| Yes | 0.586 | 0.298 | 1.153 | 2.3912/0.1220 |
| No (ref. 1.0) |  |  |  |  |
| **Pollen-allergic** |  |  |  |  |
| Yes | 0.936 | 0.476 | 1.841 | 0.0369/0.8477 |
| No (ref 1.0) |  |  |  |  |
| **Mould-allergic** |  |  |  |  |
| Yes | 0.739 | 0.349 | 1.567 | 0.6228/0.4300 |
| No (ref. 1.0) |  |  |  |  |
| **Fur allergy (cat, dog *ect.*)** |  |  |  |  |
| Yes | 0.589 | 0.286 | 1.210 | 2.0757/0.1497 |
| No (ref. 1.0) |  |  |  |  |
| **Other allergen-allergic** |  |  |  |  |
| Yes | 0.728 | 0.373 | 1.418 | 0.8723/0.3503 |
| No (ref. 1.0) |  |  |  |  |

1. Adjustment for age, sex.

2. Adjustment for age, sex, weight, height.

3. Adjustment for age, sex, weight, height, BMI, region of residence, education, occupation, smoking and drinking.

4. Adjustment for age, sex, weight, height, BMI, region of residence, education, occupation, smoking and drinking, comorbidity, medication.

5. Adjustment for age, sex, weight, height, BMI, region of residence, education, occupation, smoking and drinking, comorbidity, medication, vaccination and previous infection.

**Table S5.** STROBE Statement—Checklist of items that should be included in reports of *cohort studies*

|  | **Item No** | **Recommendation** | **Page No** |
| --- | --- | --- | --- |
| **Title and abstract** | 1 | (*a*) Indicate the study’s design with a commonly used term in the title or the abstract | 3 |
|  |  | (*b*) Provide in the abstract an informative and balanced summary of what was done and what was found | 3 |
| **Introduction** | | | |
| Background/rationale | 2 | Explain the scientific background and rationale for the investigation being reported | 5 |
| Objectives | 3 | State specific objectives, including any prespecified hypotheses | 5 |
| **Methods** | | | |
| Study design | 4 | Present key elements of study design early in the paper | 7 |
| Setting | 5 | Describe the setting, locations, and relevant dates, including periods of recruitment, exposure, follow-up, and data collection | 7 |
| Participants | 6 | (*a*) Give the eligibility criteria, and the sources and methods of selection of participants. Describe methods of follow-up | 7 |
|  |  | (*b*) For matched studies, give matching criteria and number of exposed and unexposed | NA |
| Variables | 7 | Clearly define all outcomes, exposures, predictors, potential confounders, and effect modifiers. Give diagnostic criteria, if applicable | 7 |
| Data sources/ measurement | 8* | For each variable of interest, give sources of data and details of methods of assessment (measurement). Describe comparability of assessment methods if there is more than one group | 7 |
| Bias | 9 | Describe any efforts to address potential sources of bias | 8 |
| Study size | 10 | Explain how the study size was arrived at | 8 |
| Quantitative variables | 11 | Explain how quantitative variables were handled in the analyses. If applicable, describe which groupings were chosen and why | 8 |
| Statistical methods | 12 | (*a*) Describe all statistical methods, including those used to control for confounding | 8 |
|  |  | (*b*) Describe any methods used to examine subgroups and interactions | 8 |
|  |  | (*c*) Explain how missing data were addressed | 8 |
|  |  | (*d*) If applicable, explain how loss to follow-up was addressed | NA |
|  |  | (*e*) Describe any sensitivity analyses | 8-9 |
| **Results** | | |  |
| Participants | 13* | (a) Report numbers of individuals at each stage of study—eg numbers potentially eligible, examined for eligibility, confirmed eligible, included in the study, completing follow-up, and analysed | 9 |
|  |  | (b) Give reasons for non-participation at each stage | NA |
|  |  | (c) Consider use of a flow diagram | Figure 1 |
| Descriptive data | 14* | (a) Give characteristics of study participants (eg demographic, clinical, social) and information on exposures and potential confounders | Table1 and Supplementary Material |
|  |  | (b) Indicate number of participants with missing data for each variable of interest | NA |
|  |  | (c) Summarise follow-up time (eg, average and total amount) | NA |
| Outcome data | 15* | Report numbers of outcome events or summary measures over time | 9 |

| Main results | 16 | (*a*) Give unadjusted estimates and, if applicable, confounder-adjusted estimates and their precision (eg, 95% confidence interval). Make clear which confounders were adjusted for and why they were included | 10-11 |
| --- | --- | --- | --- |
|  |  | (*b*) Report category boundaries when continuous variables were categorized | 10-11 |
|  |  | (*c*) If relevant, consider translating estimates of relative risk into absolute risk for a meaningful time period | NA |
| Other analyses | 17 | Report other analyses done—eg analyses of subgroups and interactions, and sensitivity analyses | 10-11 |
| **Discussion** | | | |
| Key results | 18 | Summarise key results with reference to study objectives | 12 |
| Limitations | 19 | Discuss limitations of the study, taking into account sources of potential bias or imprecision. Discuss both direction and magnitude of any potential bias | 15 |
| Interpretation | 20 | Give a cautious overall interpretation of results considering objectives, limitations, multiplicity of analyses, results from similar studies, and other relevant evidence | 15 |
| Generalisability | 21 | Discuss the generalisability (external validity) of the study results | 15 |
| **Other information** | | | |
| Funding | 22 | Give the source of funding and the role of the funders for the present study and, if applicable, for the original study on which the present article is based | 22 |

*Give information separately for exposed and unexposed groups.

**Note:** An Explanation and Elaboration article discusses each checklist item and gives methodological background and published examples of transparent reporting. The STROBE checklist is best used in conjunction with this article (freely available on the Web sites of PLoS Medicine at http://www.plosmedicine.org/, Annals of Internal Medicine at http://www.annals.org/, and Epidemiology at http://www.epidem.com/). Information on the STROBE Initiative is available at http://www.strobe-statement.org.

**Table S6. Questionnaire of the study of Risk and Protective Factors for Coronavirus Disease 2019 (COVID-19).**

-------------------------------------------------------------------------------------------------------

Dear Sir/Madam,

We are a research team from the Beijing Tongren Hospital, Capital Medical University. In order to study the Risk and Protective Factors for Coronavirus Disease 2019 (COVID-19), we conducted a questionnaire survey. This questionnaire survey is absolutely anonymous, definitely it will not have any negative influence on your life. The contents and data involved in the questionnaire will only be used for this study, and any personal information will be kept strictly confidential. If you agree, it will take 6-10 minutes to complete the questionnaire.
Thank you for your sincere answer and help!

**Basic Information**

1. **Sex:**

○ Male ○ Female

**2. Age: (Fill in the blank)**

___________________________

**3. Hight: _____________cm (Fill in the blank)**

**Weight: _____________kg (Fill in the blank)**

**4. Smoking history**

○Never ○Sometimes (Link to 4.1) ○ Ever, I have quit.

**4.1. How many cigarettes do you smoke every day?** (Link to 4.2)

○Less than 5 / day ○5 ~ 10 / day ○ 10 ~ 20 / day

○20 ~ 40 / day ○more than 40 / day

**4.2. How many years have you been smoking?**

○1 year

○ 2 ～ 5 years

○ 6 ～ 10 years

○10 ～ 20 years

○ 20 ～ 30 years

○30 ～ 50 years

○more than 50 years

**5. Have you been drinking?**

○Never

○Sometimes (Link to 5.1)

○ Ever, I have quit.

**5.1. How many alcohols do your intake every day?** (Estimate based on the amount of white wine consumed.) (Link to 5.2)

○Never

○Occasionally

○Have drinking history, but have quit drinking

○Less than 50 mL / day

○50 mL ～ 100 mL / day

○100 mL ～ 250 mL day

○More than 250mL / day

**5.2. How many years have you been drinking?**

○ 1 year

○ 2 ～ 5 years

○ 6 ～ 10 years

○10 ～ 20 years

○ 20 ～ 30 years

○ 30 ～ 50 years

○ More than 50 years

**6. Where is your residence? (Fill in the blank)**

___________________________

**7. The area you live (multiple options)**

🞎 City

🞎 Township

🞎 Village

**8. Education**

○ Below junior

○ Junior

○ Senior

○ College

○ Postgraduate and higher

**9. What is your job?**

○ Restaurant workers

○ Logistics workers

○ Other service industry workers (e.g. healthcare workers or hospital staff)

○ Workers in general

○ Teachers

○ Student

○ Farmer

○ Businessman

○ Official

○ Individual Businessman

○ Freelancer

○ Others

**10. Have you experienced symptoms such as sneezing, runny nose, itchiness or nasal congestion when exposed to allergens such as pollen or dust, pet hair in the past 12 months?**

○Yes

○No

**11. Have you had a blood test or skin allergy test?**

○Yes

○No

**12. Blood or skin allergy test results:**

○Negative

○Positive (link to the secondary options) (multiple options)

🞎 Pollen from trees such as poplars, willows, or birches

🞎 Broomweed and other autumn weeds produce pollen or flowers from other plants

🞎 Dust mite allergy

🞎 Mold allergy

🞎 Pet hair allergy

🞎 Other allergies (such as cockroaches, dust, house dust, etc.)

**13. Have you experience sneezing, runny nose, etc. when you come across temperature** **changes, irritating odors, or foods such as pepper and chili?**

○Yes

○No

**14. Have you ever suffering from other related diseases? (multiple options)**

🞎 None

🞎 Chronic rhinosinusitis

🞎 Chronic bronchitis

🞎 Chronic obstructive pulmonary disease

🞎 Skin allergies (such as hives, welts, eczema, itching/rashes, atopic dermatitis, etc.)

🞎 Allergic conjunctivitis

🞎 Food allergies

🞎 Hypertension

🞎 Coronary heart disease

🞎 Other cardiovascular diseases

🞎 Rheumatic or immune diseases

🞎 Anxiety or depression

🞎 Cerebrovascular diseases

🞎 Diagnosed with or treated for malignant tumors

**15. Have you ever been infected with COVID-19?**

○Yes (link to 15.1) ○No

**15.1 The date of your infection with COVID-19 (onset symptoms/positive nucleic acid antibody test): (Fill in the blank)**

___________________________

**16. For allergic rhinitis, nasal congestion, or related complications, do you consistently take the following treatment? (multiple options)**

🞎 None

🞎 Nasal spray corticosteroids (such as Rhinocort / Budesonide, Flixonase / Fluticasone Propionate, Nasonex / Mometasone Furoate, etc.)

🞎 Oral corticosteroids (such as Prednisone, Prednisolone tablets, etc.)

🞎 Oral anti-histamine (such as Loratadine, Cetirizine, etc.)

🞎 Anti-leukotrienes (such as Montelukast, etc.)

🞎 Inhaled corticosteroids (such as Budesonide Suspension for Inhalation, etc.)

🞎 Antibiotics

🞎 Expectorants (such as Eucalyptol, Limonene and Pinene Enteric, Sinupret Drops etc.)

🞎 Nasal irrigation (such as saline solution, etc.)

🞎 Biologics (such as Omalizumab, etc.)

🞎 Inhaled corticosteroids or combination therapies containing bronchodilators (such as Seretide, Flixotide Evohaler, Symbicort Turbuhaler, etc.)

🞎 Theophylline

🞎 Long-acting anticholinergic agents (such as Atrovent, etc)

🞎 Others

**17. Please describe your nasal symptoms before you contracted the COVID-19.**

17.1 Nasal congestion

○None ○Mild ○Moderate ○Severe
17.2 Nasal itching and sneezing

○None ○Mild ○Moderate ○Severe
17.3 Runny nose - mucous or purulent discharge

○None ○Mild ○Moderate ○Severe
17.4 Runny nose - clear discharge

○None ○Mild ○Moderate ○Severe

17.5 Sneezing

○None ○Mild ○Moderate ○Severe
17.6 Loss of smell or taste

○None ○Mild ○Moderate ○Severe
17.7 Nasal dryness and pain

○None ○Mild ○Moderate ○Severe
17.8 Nasal bleeding

○None ○Mild ○Moderate ○Severe

**18. Please describe your ear symptoms before you contracted the COVID-19.**

18.1 Ear congestion

○None ○Mild ○Moderate ○Severe
18.2 Earache

○None ○Mild ○Moderate ○Severe
18.3 Decreased hearing

○None ○Mild ○Moderate ○Severe
18.4 Tinnitus

○None ○Mild ○Moderate ○Severe
18.5 Dizziness

○None ○Mild ○Moderate ○Severe
18.6 Vertigo

○None ○Mild ○Moderate ○Severe

**19. Please describe your other symptoms before you contracted the COVID-19.**

19.1Headache

○None ○Mild ○Moderate ○Severe
19.2 Sore throat

○None ○Mild ○Moderate ○Sever
19.3 Dry/itchy throat

○None ○Mild ○Moderate ○Severe
19.4 Cough

○None ○Mild ○Moderate ○Severe
19.5 Phlegm

○None ○Mild ○Moderate ○Severe
19.6 Hoarseness

○None ○Mild ○Moderate ○Severe

19.7 Chest distress

○None ○Mild ○Moderate ○Severe
19.8 Shortness of breath

○None ○Mild ○Moderate ○Severe
19.9 Pressure or pain in the face or eye area

○None ○Mild ○Moderate ○Severe

19.10 Loss of taste

○None ○Mild ○Moderate ○Severe

19.11 Skin sensitivity (such as hives, welts, rashes, itching/rashes, eczema, etc.)

○None ○Mild ○Moderate ○Severe

19.12 Fatigue or feeling of tiredness

○None ○Mild ○Moderate ○Severe

19.13 Muscle discomfort or pain diarrhea

○None ○Mild ○Moderate ○Severe

19.14 Diarrhea

○None ○Mild ○Moderate ○Severe

19.15 Nausea or vomiting

○None ○Mild ○Moderate ○Severe

**20. Have you taken an antigen / RNA test for the Covid-19 infection?**

○ Nucleic acid test positive

○ Antigen test positive

○ Both positive of nucleic acid and antigen tests

○ Negative

○ Not tested

**21. How many times have you take vaccines against COVID-19？**

○ Once

○ Twice

○ Three times

○ Four times

○ Never

**22. The time interval between last vaccination and onset of COVID-19 symptoms？**

○ Unvaccinated

○ Less than 1 month

○ 1 ~ 3 months

○ 3 ~ 6 months

○ 6 ~ 12 months

○ 6 ~ 12 months

○ 1 ~ 2 years

○ More than 2 years

**23. Have you been diagnosed with pneumonia as a result of this COVID-19 infection?**

○ Unclear

○ Yes

○ No

**24. Have you ever taken a chest CT or X-ray?**

○ None

○ Yes, pneumonia

○ Yes, normal

**25. The duration of the initial symptoms to recovery (antigen / RNA conversion to negative, or disappearance of main symptoms)？**

○ 1 day

○ 2 days

○ 3 days

○ 4 days

○ 5 days

○ 6 days

○ 7 days

○ 8 days

○ 9 days

○ 10 days

○ 11 days

○ 12 days

○ 13 days

○ 14 days

○ more than 14 days

**26. How many times you have had COVID in the past?**

○ Never

○ Once

○ Twice

○ More than twice

**27. Are you feeling feverish and what is your highest temperature (in °C)?**

○ None

○ 37.3 ~ 38.0 °C

○ 38.1 ~ 39.0 °C

○ Higher than 39.0 °C

**28. How long has the fever lasted?**

○ None

○ Half a day

○ 1 day

○ 2 days

○ 3 days

○ 4 days

○ 5 days

○ More than 5 days

**29. Please describe your nasal symptoms after you infected the COVID-19.**

29.1 Nasal congestion

○None ○Mild ○Moderate ○Severe
29.2 Nasal itching and sneezing

○None ○Mild ○Moderate ○Severe
29.3 Runny nose - mucous or purulent discharge

○None ○Mild ○Moderate ○Severe
29.4 Runny nose - clear discharge

○None ○Mild ○Moderate ○Severe
29.5 Sneezing

○None ○Mild ○Moderate ○Severe

29.6 Loss of smell or taste

○None ○Mild ○Moderate ○Severe
29.7 Nasal dryness and pain

○None ○Mild ○Moderate ○Severe
29.8 Nasal bleeding

○None ○Mild ○Moderate ○Severe
**30. The duration of nasal symptoms after infecting COVID-19？**

30.1 Nasal congestion

○None ○1～2 days ○3～4 days ○5～7 days ○1～2 weeks ○more than 2 weeks

30.2 Nasal itching and sneezing

○None ○1～2 days ○3～4 days ○5～7 days ○1～2 weeks ○more than 2 weeks

30.3 Runny nose - mucous or purulent discharge

○None ○1～2 days ○3～4 days ○5～7 days ○1～2 weeks ○more than 2 weeks

30.4 Runny nose - clear discharge

○None ○1～2 days ○3～4 days ○5～7 days ○1～2 weeks ○more than 2 weeks

30.5 Sneezing

○None ○1～2 days ○3～4 days ○5～7 days ○1～2 weeks ○more than 2 weeks

30.6 Loss of smell or taste

○None ○1～2 days ○3～4 days ○5～7 days ○1～2 weeks ○more than 2 weeks

30.7 Nasal dryness and pain

○None ○1～2 days ○3～4 days ○5～7 days ○1～2 weeks ○more than 2 weeks

30.8 Nasal bleeding

○None ○1～2 days ○3～4 days ○5～7 days ○1～2 weeks ○more than 2 weeks

**31. Please describe your ear symptoms after you contracted the COVID-19.**

31.1 Ear congestion

○None ○Mild ○Moderate ○Severe
31.2 Earache

○None ○Mild ○Moderate ○Severe
31.3 Decreased hearing

○None ○Mild ○Moderate ○Severe
31.4 Tinnitus

○None ○Mild ○Moderate ○Severe
31.5 Dizziness

○None ○Mild ○Moderate ○Severe
31.6 Vertigo

○None ○Mild ○Moderate ○Severe

**32. The duration of ear symptoms after infecting COVID-19？**

32.1 Ear congestion

○None ○1～2 days ○3～4 days ○5～7 days ○1～2 weeks ○more than 2 weeks

32.2 Earache

○None ○1～2 days ○3～4 days ○5～7 days ○1～2 weeks ○more than 2 weeks

32.3 Decreased hearing

○None ○1～2 days ○3～4 days ○5～7 days ○1～2 weeks ○more than 2 weeks
32.4 Tinnitus

○None ○1～2 days ○3～4 days ○5～7 days ○1～2 weeks ○more than 2 weeks

32.5 Dizziness

○None ○1～2 days ○3～4 days ○5～7 days ○1～2 weeks ○more than 2 weeks
32.6 Vertigo

○None ○1～2 days ○3～4 days ○5～7 days ○1～2 weeks ○more than 2 weeks

**33. Please describe your other symptoms after you contracted the COVID-19.**

33.1 Headache

○None ○Mild ○Moderate ○Severe
33.2 Sore throat

○None ○Mild ○Moderate ○Sever
33.3 Dry/itchy throat

○None ○Mild ○Moderate ○Severe
33.4 Cough

○None ○Mild ○Moderate ○Severe
33.5 Phlegm

○None ○Mild ○Moderate ○Severe
33.6 Hoarseness

○None ○Mild ○Moderate ○Severe
33.7 Chest distress

33.8 Shortness of breath

○None ○Mild ○Moderate ○Severe
33.9 Pressure or pain in the face or eye area

○None ○Mild ○Moderate ○Severe

33.10 Loss of taste

○None ○Mild ○Moderate ○Severe

33.11 Skin sensitivity (such as hives, welts, rashes, itching/rashes, eczema, etc.)

○None ○Mild ○Moderate ○Severe

33.12 Fatigue or feeling of tiredness

○None ○Mild ○Moderate ○Severe

33.13 Muscle discomfort or pain diarrhea

○None ○Mild ○Moderate ○Severe

33.14 Diarrhea

○None ○Mild ○Moderate ○Severe

33.15 Nausea or vomiting

○None ○Mild ○Moderate ○Severe

**34. The duration of other symptoms after infecting COVID-19？**

34.1 Headache

○None ○1～2 days ○3～4 days ○5～7 days ○1～2 weeks ○more than 2 weeks

34.2 Sore throat

○None ○1～2 days ○3～4 days ○5～7 days ○1～2 weeks ○more than 2 weeks

34.3 Dry/itchy throat

○None ○1～2 days ○3～4 days ○5～7 days ○1～2 weeks ○more than 2 weeks
34.4 Cough

○None ○1～2 days ○3～4 days ○5～7 days ○1～2 weeks ○more than 2 weeks

34.5 Phlegm

○None ○1～2 days ○3～4 days ○5～7 days ○1～2 weeks ○more than 2 weeks
34.6 Hoarseness

○None ○1～2 days ○3～4 days ○5～7 days ○1～2 weeks ○more than 2 weeks

34.7 Chest distress

○None ○1～2 days ○3～4 days ○5～7 days ○1～2 weeks ○more than 2 weeks

34.8 Shortness of breath

○None ○1～2 days ○3～4 days ○5～7 days ○1～2 weeks ○more than 2 weeks

34.9 Pressure or pain in the face or eye area

○None ○1～2 days ○3～4 days ○5～7 days ○1～2 weeks ○more than 2 weeks

34.10 Loss of taste

○None ○1～2 days ○3～4 days ○5～7 days ○1～2 weeks ○more than 2 weeks

34.11 Skin sensitivity (such as hives, welts, rashes, itching/rashes, eczema, etc.)

○None ○1～2 days ○3～4 days ○5～7 days ○1～2 weeks ○more than 2 weeks

34.12 Fatigue or feeling of tiredness

○None ○1～2 days ○3～4 days ○5～7 days ○1～2 weeks ○more than 2 weeks

34.13 Muscle discomfort or pain diarrhea

○None ○1～2 days ○3～4 days ○5～7 days ○1～2 weeks ○more than 2 weeks

34.14 Diarrhea

○None ○1～2 days ○3～4 days ○5～7 days ○1～2 weeks ○more than 2 weeks

34.15 Nausea or vomiting

○None ○1～2 days ○3～4 days ○5～7 days ○1～2 weeks ○more than 2 weeks

**35. For the symptoms caused by COVID-19, have you taken the following treatment measures? (multiple options)**

🞎 No treatment

🞎 eat-clearing and detoxicating medicine

🞎 Antipyretic Analgesics

🞎 Antitussives and Expectorants

🞎 Aerosol inhalant

🞎 inhaled corticosteroids or compound drugs

🞎 Antibiotics

🞎 Medicines to Improve Gastrointestinal Symptoms

🞎 Effective Drugs for COVID-19

🞎 Others

**SUPPLEMENTARY FIGURES**

**
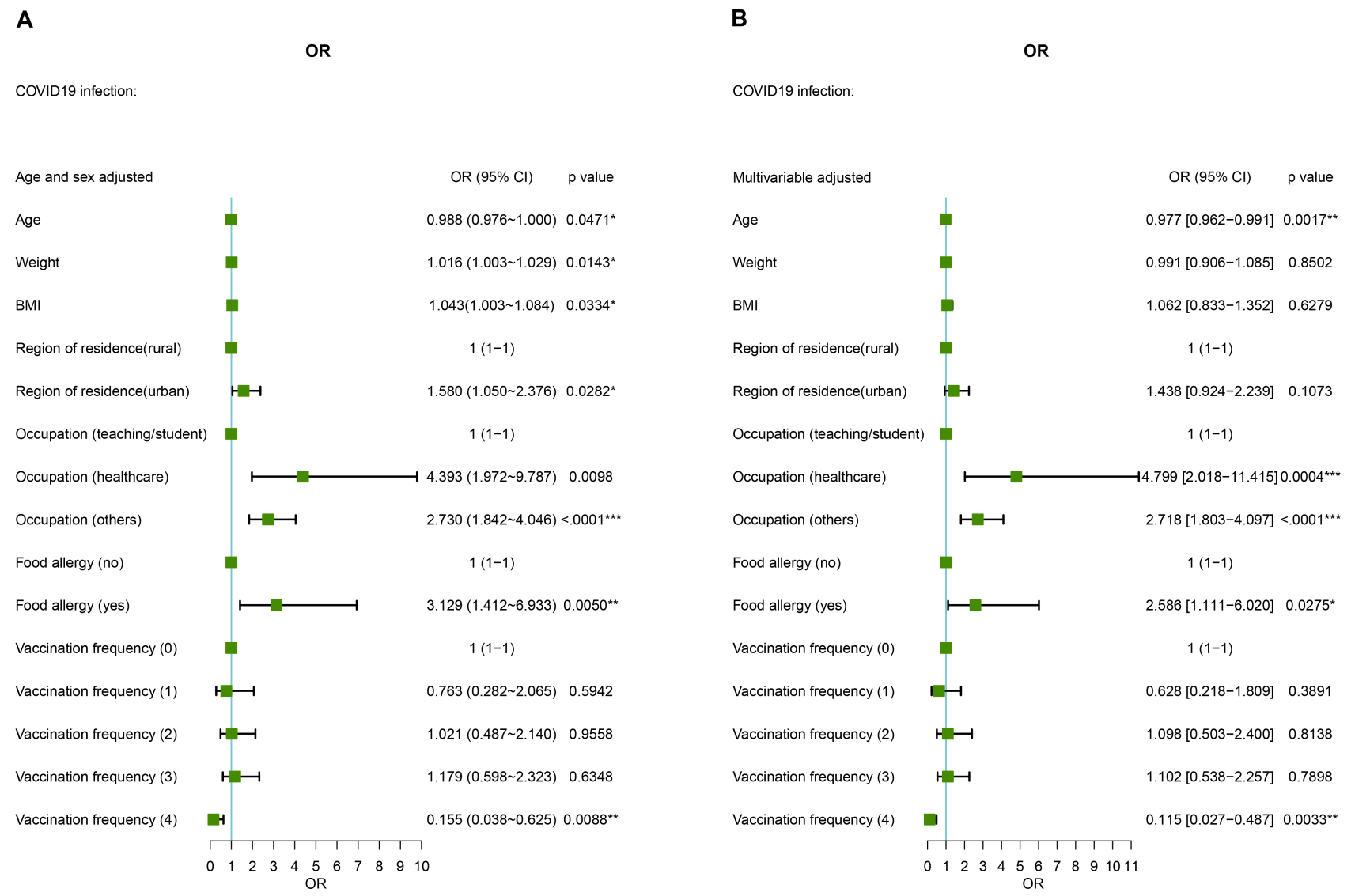
**

**Figure S1. Age, sex adjusted (A) and Multiple-adjusted (B) ORs for COVID19 infection (with PCR test positive or antigen detection positive or both of two tests are positive) associated with risk factors in AR patients.**

**
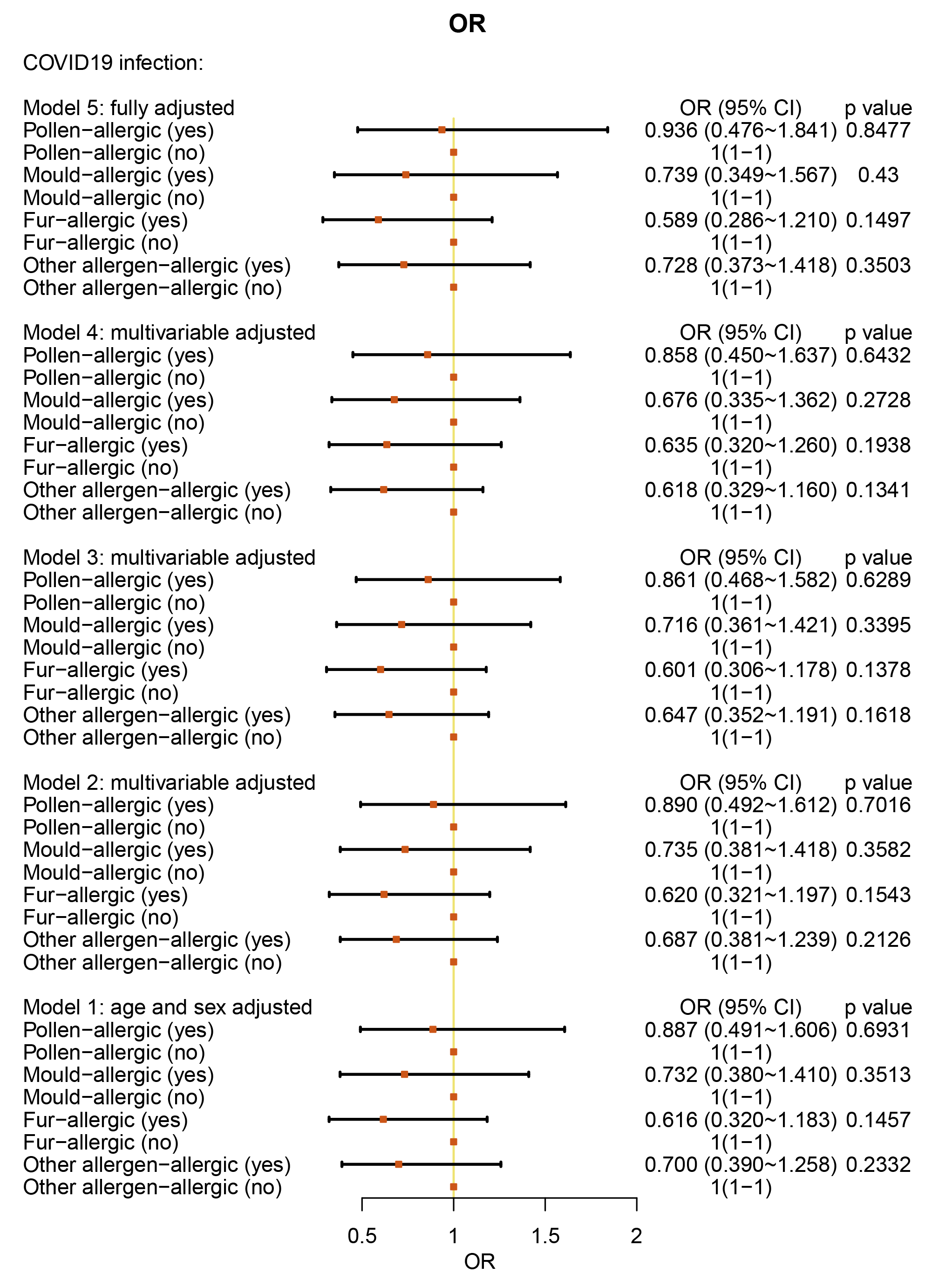
Figure S2. Allergen specific (except mite) multiple-adjusted ORs for COVID19 infection associated with risk factors in AR patients (with allergen testing results)(n=237).**

**
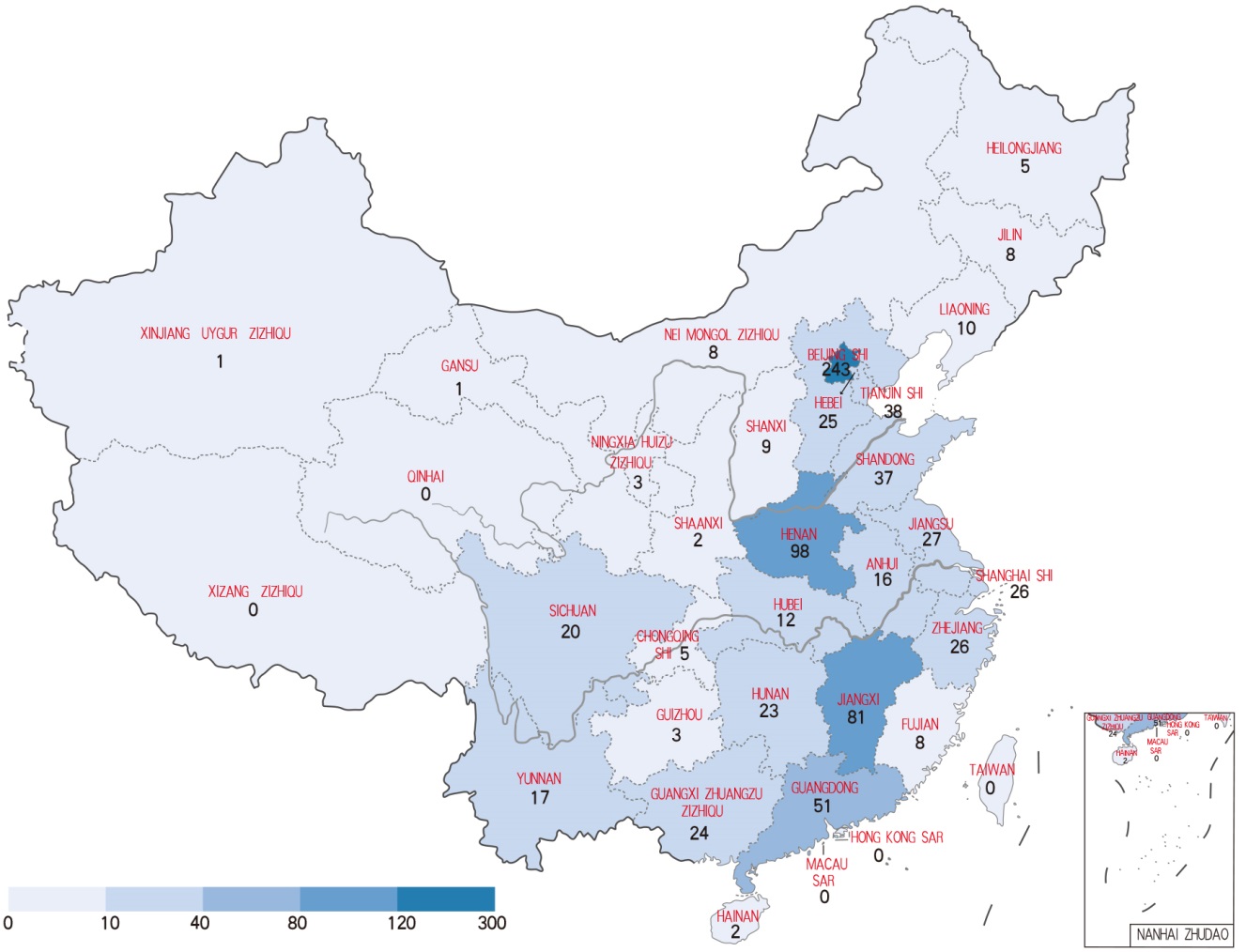
**

**Figure S3.** The geographical distribution of participants (n=829, 1 missing geographical record). Legend in the bottom indicates the number of participants.
